# The interplay of climate, intervention and imported cases as determinants of the 2014 dengue outbreak in Guangzhou

**DOI:** 10.1371/journal.pntd.0005701

**Published:** 2017-06-22

**Authors:** Qu Cheng, Qinlong Jing, Robert C. Spear, John M. Marshall, Zhicong Yang, Peng Gong

**Affiliations:** 1 Ministry of Education Key Laboratory for Earth System Modeling, Department of Earth System Science, Tsinghua University, Beijing, People’s Republic of China; 2 Department of Medical Statistics and Epidemiology, School of Public Health, Sun Yat-Sen University, Guangzhou, Guangdong, People’s Republic of China; 3 Department of Infectious Diseases, Guangzhou Center for Disease Control and Prevention, Guangzhou, Guangdong, People’s Republic of China; 4 Environmental Health Sciences, School of Public Health, University of California, Berkeley, Berkeley, California, United States of America; 5 Division of Biostatistics and Epidemiology, School of Public Health, University of California, Berkeley, Berkeley, California, United States of America; 6 Guangzhou Center for Disease Control and Prevention, Guangzhou, Guangdong, People’s Republic of China; 7 Key Laboratory of Tropical Disease Control (Sun Yat-sen University), Ministry of Education, Guangzhou, Guangdong, People’s Republic of China; 8 Joint Center for Global Change Studies, Beijing, People’s Republic of China; Sanofi Pasteur, FRANCE

## Abstract

Dengue is a fast spreading mosquito-borne disease that affects more than half of the population worldwide. An unprecedented outbreak happened in Guangzhou, China in 2014, which contributed 52 percent of all dengue cases that occurred in mainland China between 1990 and 2015. Our previous analysis, based on a deterministic model, concluded that the early timing of the first imported case that triggered local transmission and the excessive rainfall thereafter were the most important determinants of the large final epidemic size in 2014. However, the deterministic model did not allow us to explore the driving force of the early local transmission. Here, we expand the model to include stochastic elements and calculate the successful invasion rate of cases that entered Guangzhou at different times under different climate and intervention scenarios. The conclusion is that the higher number of imported cases in May and June was responsible for the early outbreak instead of climate. Although the excessive rainfall in 2014 did increase the success rate, this effect was offset by the low initial water level caused by interventions in late 2013. The success rate is strongly dependent on mosquito abundance during the recovery period of the imported case, since the first step of a successful invasion is infecting at least one local mosquito. The average final epidemic size of successful invasion decreases exponentially with introduction time, which means if an imported case in early summer initiates the infection process, the final number infected can be extremely large. Therefore, dengue outbreaks occurring in Thailand, Singapore, Malaysia and Vietnam in early summer merit greater attention, since the travel volumes between Guangzhou and these countries are large. As the climate changes, destroying mosquito breeding sites in Guangzhou can mitigate the detrimental effects of the probable increase in rainfall in spring and summer.

## Author summary

An unprecedented dengue outbreak occurred in Guangzhou, 2014, with 38,036 reported cases in contrast to 73,179 cases in all of mainland China from 1990 to 2015. In an earlier analysis using a deterministic model, we concluded the early timing of local transmission to be the most important determinant of this outbreak. Here we use a stochastic model to explore the reasons why the outbreak happened earlier in 2014. Our results identified the higher number of imported cases in May and June to be the most probable explanation. Based on the investigation of the determinants of success rate and final epidemic size, this work provides suggestions for reducing dengue outbreak potential and epidemic size in the future. More attention should be paid to imported case detection and vector control measures in early summer, because this is the time when successful invasion can result in high incidence of infection and the success rate of each imported case begins to rise. Destroying mosquito breeding sites can reduce the maximum water level of the system and attenuate the role played by climate. In addition, interventions within 10 days after the introduction of imported cases is still effective in preventing further transmission.

## Introduction

As the most important mosquito-borne disease globally, the incidence of dengue has increased 30-fold in the past 50 years [1]. In 2013 alone, dengue was responsible for 576,900 years of premature life lost and 1.14 million disability-adjusted life-years globally [2]. However, dengue was not a serious concern in mainland China before 2014. There were only 73,179 cases from 1990 to 2015, among which 47,056 (64.3%) occurred in 2014, and 80.8 percent of these cases were contributed by Guangzhou [3]. Because of its warm and wet climate, as well as the close ties with dengue endemic Southeast Asian countries, Guangzhou has a high risk of local dengue transmission. The introduction of dengue virus (DENV) by imported cases is required annually for local epidemics in Guangzhou, since the dengue virus cannot disseminate to the salivary glands of *Aedes albopictus* when temperature is below 18°C [4] and adult mosquitoes rarely survive the winter.

Many studies have attempted to explain the unprecedented dengue outbreak in Guangzhou, 2014. Possible explanations include more imported cases, higher mosquito abundance due to a more favorable climate, higher larval development and adult survival rates caused by urbanization, and lack of diagnostic experience [5, 6]. The result of our earlier analysis using a deterministic mathematical model identified the early timing of local transmission and climate as the most important determinants of the large final epidemic size (FES) [7], but the model could not explain why local transmission occurred earlier that year. Because a stochastic model can produce different results every run under the same conditions, an imported case can lead to local transmission in some runs but not so in others. Therefore, a stochastic model can shed more light on the outbreak probability and can provide greater insight into the various explanations of earlier local transmission, such as a more favorable climate for mosquito growth and more imported cases. Clearly, understanding the reasons underlying the early and large outbreak can help to prevent dengue outbreaks in the future, and reduce potential economic and health impacts.

Stochastic models have been widely used to study the invasion of a disease into non-endemic areas, such as dengue virus transmitted by *Ae*. *aegypti* mosquitoes [8], dengue virus in Madeira [9], and Chikungunya virus in the United States [10]. However, none of these models considered the importance of water availability in restricting the environmental carrying capacity and density-dependent rates.

In this paper, we simplify our deterministic model by leaving out vertical transmission from infected adults to eggs since it was found to be unimportant in the earlier analysis [7], and then extend it to incorporate stochastic effects. A hybrid deterministic/stochastic algorithm originally used in simulating chemical reactions [11–13] is used to simulate the dengue transmission dynamics. This algorithm can take advantage of the low computational burden of deterministic models while still incorporating stochastic effects. Six different scenarios are constructed to explore the determinants of early outbreak in 2014 and the effectiveness of interventions (insecticide spraying and pooled water removal) in reducing dengue transmission risk. We also address the determinants of the outbreak size and FES, as well as the implications for prediction and prevention.

## Methods

### Ethics statement

The Ethics Committee of the Guangzhou Center for Disease Control and Prevention reviewed and approved this study. All patient data was made anonymous prior to the analysis.

### Study area

**Fig 1:**
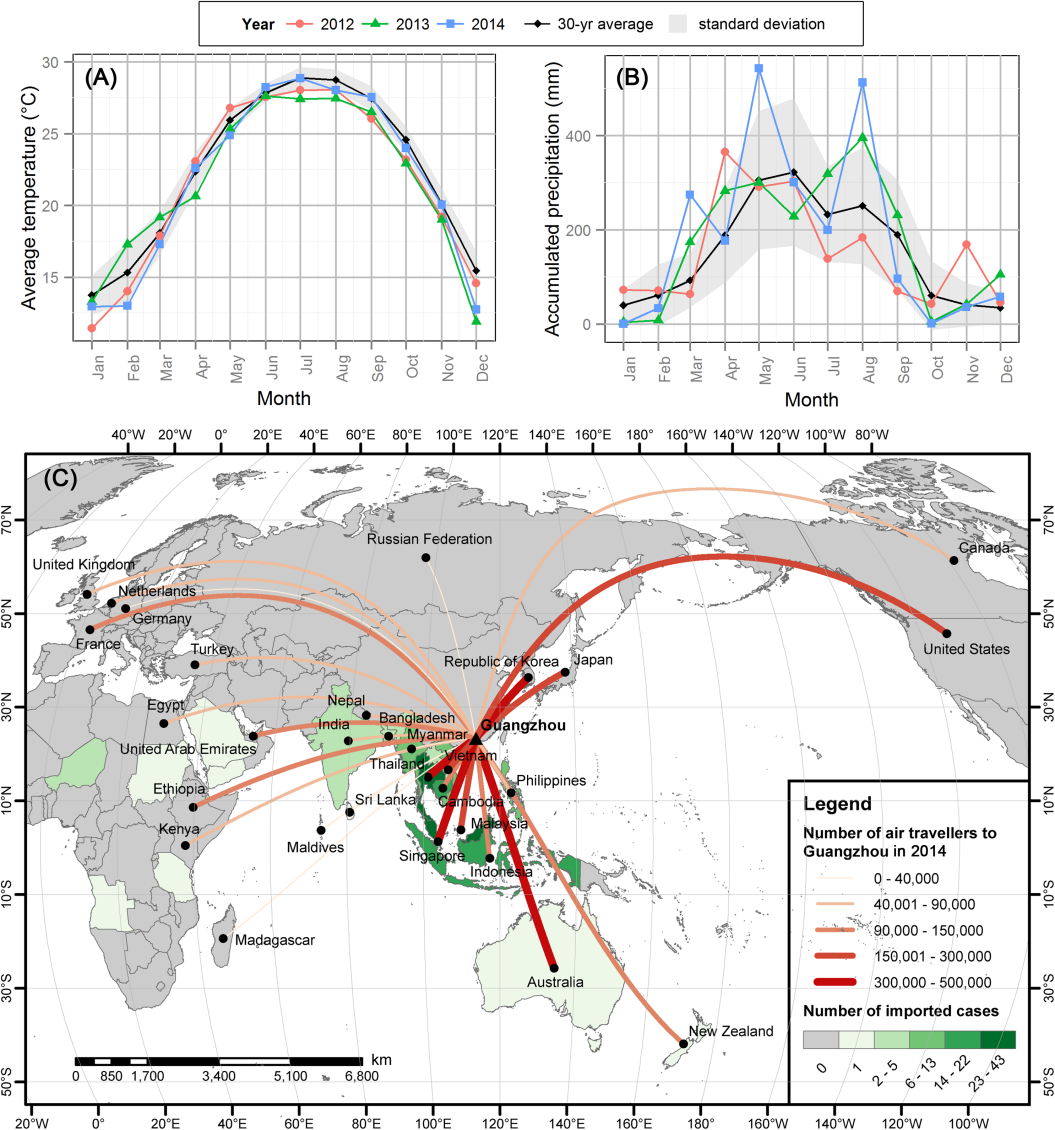
Climate in Guangzhou and the number of direct air travelers to Guangzhou in 2014. (A) Monthly mean temperature and (B) accumulated precipitation for 2012 to 2014, and 30-yr average (1985–2014). (C) Number of direct air travelers from different countries to Guangzhou in 2014 and the total number of imported cases from different countries from 2001 to 2015.

Guangzhou, with a population of 13.1 million at the end of 2014 [14], is the largest city in South China. The climate is characterized by warm and humid summers and mild and dry winters. The climate data downloaded from China Meteorological Data Sharing Service System (CMDSSS) indicates that the annual mean temperature from 1985 to 2014 was 22.4°C. January had the lowest average temperature of 13.7°C, while July and August had the highest at 28.9 and 28.7°C, respectively (Fig 1A). The average annual accumulated precipitation was 1,821 mm, of which 1,490 mm (81.9%) occurred between April and September (Fig 1B). The warm and wet summer is favorable for the growth of *Ae*. *albopictus*, the sole vector of dengue transmission in Guangzhou [15].

Because low temperature is unable to support virus replication in *Ae*. *albopictus* in the winter [4], the occurrence of dengue epidemics in Guangzhou depends highly on imported cases from surrounding endemic countries, such as Thailand, Singapore, Malaysia, Philippines, Vietnam, Cambodia, and Indonesia. Serving as a transportation hub for these countries increases the outbreak risk of Guangzhou further (Fig 1C).

### Data

We collected daily reported dengue case numbers in 2013 and 2014 from the Guangzhou Center for Disease Control and Prevention (Guangzhou CDC). These data are also available from the Health and Family Planning Commission of Guangdong Province (http://www.gdwst.gov.cn/) in the transmission season. Both passive and active surveillance systems are used in China. Health-care providers are required to diagnose the disease according to the National Diagnostic Criteria for Dengue Fever (WS216-2008) and then report the new cases to the web-based National Notifiable Infectious Disease Reporting Information System within 24 hours. After the confirmation of a case, active detection is initiated and conducted by various means, such as interviewing residents in the same community, checking employee attendance records in the same work place, and checking the outpatient medical records in nearby health facilities [16]. Cases are categorized further into imported and indigenous cases based on the travel and mosquito biting history. Imported cases are those who have traveled to dengue endemic regions and been bitten by mosquitoes less than 15 days before the symptom onset [6]. This dataset was used to calibrate the deterministic model.

Entomological surveillance data, including the Breteau index (BI) and mosquito ovitrap index (MOI) for 2013 and 2014, were also obtained from Guangzhou CDC. BI is a representation of larva abundance, which is defined as the number of water containers infested with larva per 100 houses inspected, while MOI is a proxy for adult abundance defined as the percentage of positive ovitraps in a specific area. The daily temperature, precipitation and evaporation data for Guangzhou from 1985 to 2014 were downloaded from CMDSSS (http://data.cma.cn/). The 30-yr daily average climatic factors were calculated to represent a typical year and were used in *Scenario 2014 to Avg* described later. Climate datasets were used as inputs to the model.

Human population data were extracted from the Guangdong Statistical Yearbook [17–19] to estimate the birth and death rates, which were treated as known parameters in the deterministic model. In addition, the initial value for the susceptible human population in the model was set to the population at the end of 2011, because we assumed that all residents were susceptible to dengue virus infection since no big outbreaks had occurred in Guangzhou from 1978.

To investigate the tourist exchange between Guangzhou and dengue endemic countries, we also extracted the number of direct air travelers between Guangzhou and different countries from on-flight origin and destination (OFOD) data provided by International Civil Aviation Organization (ICAO) (https://www4.icao.int/NewDataPlus/). Since this dataset leaves out indirect air travelers or travelers by sea or by land, we also collected the number of foreign tourists staying overnight in Guangzhou and the number of tourists who traveled from Guangzhou with tour groups from the Tourism Administration of Guangzhou (http://www.gzly.gov.cn/index.html) as a complement (S1 Table).

### Model description

**Fig 2:**
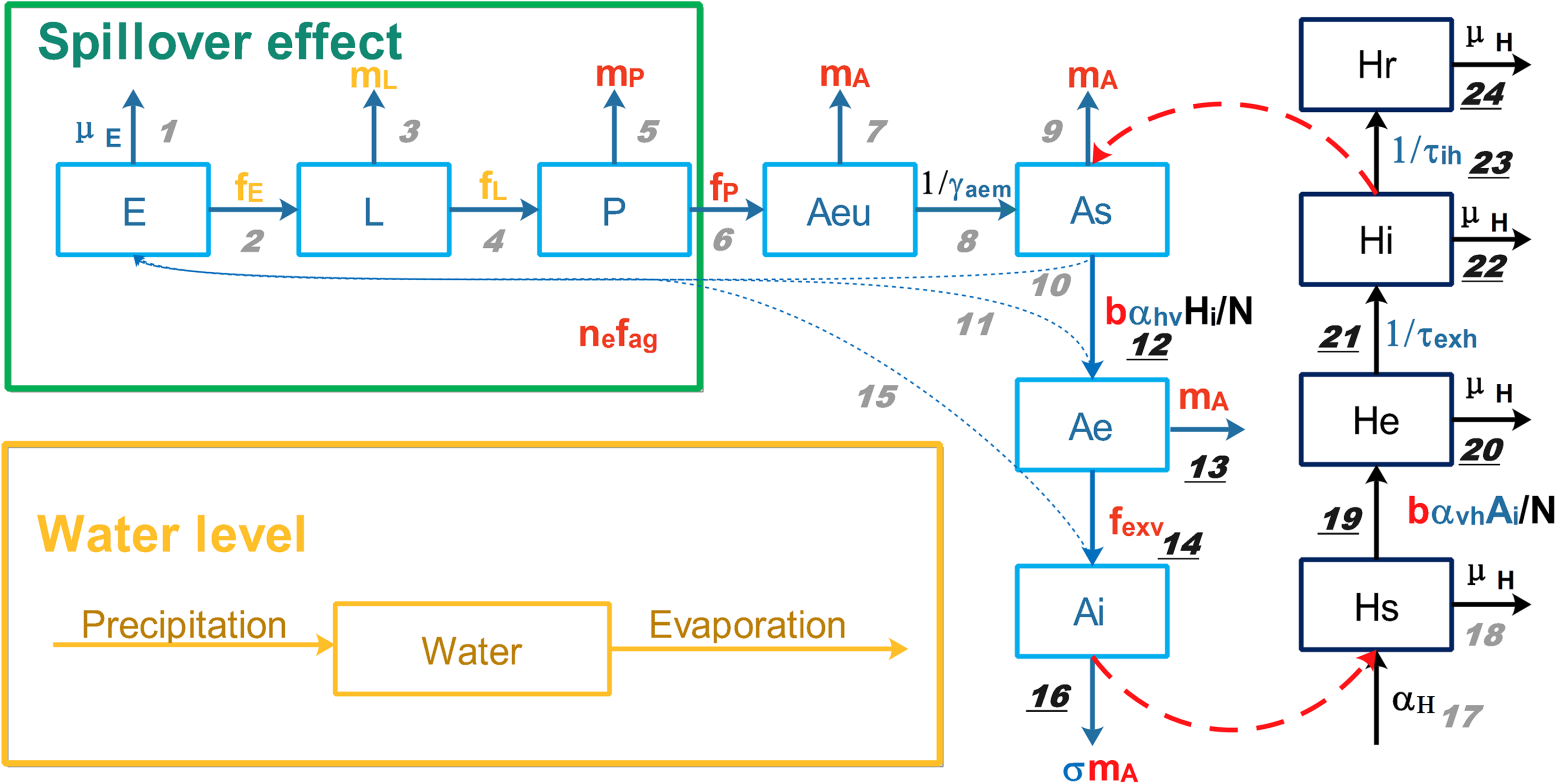
Model structure. Blue and black rectangles represent mosquito and human subpopulations, respectively. Greek letters stand for constant rates, among which the black letters can be estimated from statistical yearbooks, while the blue letters are unknown and need to be estimated in the deterministic model. English letters stand for transition functions, among which the red letters only depend on temperature, while the yellow letters depend on both temperature and water level. If a heavy rainfall occurs when the current water level is close to its maximum value, a fraction of the immature stage mosquitos will be washed out (spillover effect) [22]. Numbers are the IDs for different events. Only events with underlined IDs are modelled stochastically. More information can be found in [7] and S1 File.

To simulate the population dynamics and dengue virus infection status of both *Ae*. *albopictus* and humans, we constructed a compartment model shown in Fig 2 which is based on Ross-Macdonald model [20, 21]. Several modifications were made to the basic model to emphasis the influence of temperature and precipitation, such as modelling water level and aquatic stages explicitly, and using temperature- and density-dependent rates. The model has been described in detail elsewhere [7]. Because vertical transmission was found to be unimportant in the 2014 outbreak [7], here we removed the vertical infected egg, larva, pupa and emerging adult compartments to simplify the model structure and to reduce computational complexity. Immature life stages eggs (*E*), larvae (*L*), and pupae (*P*) were included to incorporate the effects of water availability and temperature on vector abundance. Emerging adults (*Aeu*) were also incorporated as a separate compartment because they do not bite. Then, according to infection status, the biting mosquito population was divided further into susceptible (*As*), exposed (*Ae*) and infectious (*Ai*) adults, and the human population was divided into susceptible (*Hs*), exposed (*He*), infectious (*Hi*) and recovered (*Hr*) individuals.

Transition rates in the model depend on temperature and water availability. Temperature can influence the development rate from eggs to larvae, larvae to pupae, and pupae to emerging adults (Events 2, 4 and 6 in Fig 2), the mortality rate of larvae, pupae and adults (Events 3, 5, 7, 9, 13 and 16), the biting rate of adults (Events 12 and 19), the extrinsic incubation period (EIP) of dengue virus (Event 14), and the average number of eggs and duration of each gonotrophic cycle (Events 10, 11 and 15) [23–25]. Therefore, temperature-dependent functions whose form is based on [23, 26] were used to describe these rates, and the coefficients in these functions were estimated from experiments conducted on *Ae*. *albopictus* strains from South China [25, 27]. Furthermore, the development rate from eggs to larvae, larvae to pupae, and the mortality rate of larvae also depend on the larval density (Events 2–4), which further depend on the current available water level determined by rainfall, evaporation, the minimum and maximum water level (ω_min_ and ω_max_) of the system, and interventions (eg. emptying water containers) [28]. Minimum water level represents standing waters that are difficult to evaporate, such as the water in containers with lids or in shaded areas, while the maximum water level is the upper limit of the water in the system beyond which water will overflow. Since these two parameters cannot be obtained from the literature or surveys, they were estimated by the deterministic model. More information for the model structure can be found in the S1 File.

As in the previous paper [7], we still include the arrival of imported cases, intervention, the spillover effect and diapausing eggs in the current model. The model was run from 2012 to 2014, though we only fit the model to the observed daily reported cases of 2013 and 2014. Year 2012 was included to get a reliable mosquito population for 2013 and 2014, because the initial value for eggs can only affect the mosquito abundance of the first simulated year, but not in later years [29].

### Parameterization, calibration and validation of the deterministic model

There are 19 parameters in our model and their values are highly uncertain. In addition, we are more confident about the pattern of the daily reported new cases and mosquito surveillance data than the exact value of these data on any given day. Therefore, instead of employing Markov Chain Monte Carlo (MCMC) fitting procedures common in epidemiologic mathematical modelling [9, 30], we used a parameter estimation strategy originally called regional sensitivity analysis (RSA) [31] to fit the model to the pattern of the surveillance data. The detailed process was described elsewhere [7], and only a brief description is provided here.

To calibrate the deterministic model, a broad range for each parameter was specified based on the literature or our best knowledge. Classification criteria were defined to describe whether the model output mimicked the observed timing and approximate number of reported cases at the onset, peak and end phase of the epidemics (see details for the criteria in S1 File). A Monte Carlo procedure was then followed in which random samples were drawn from the specified parameter space and used to run the mode. The output of each realization was classified into one of two subgroups “pass” or “fail” according to whether the result met all of the classification criteria. After accumulating a sufficient number of parameter vectors in the “pass” group, the characteristics of the cumulative univariate distributions were used to trim the range for each parameter to get a smaller parameter subspace with a higher passing rate.

**Fig 3:**
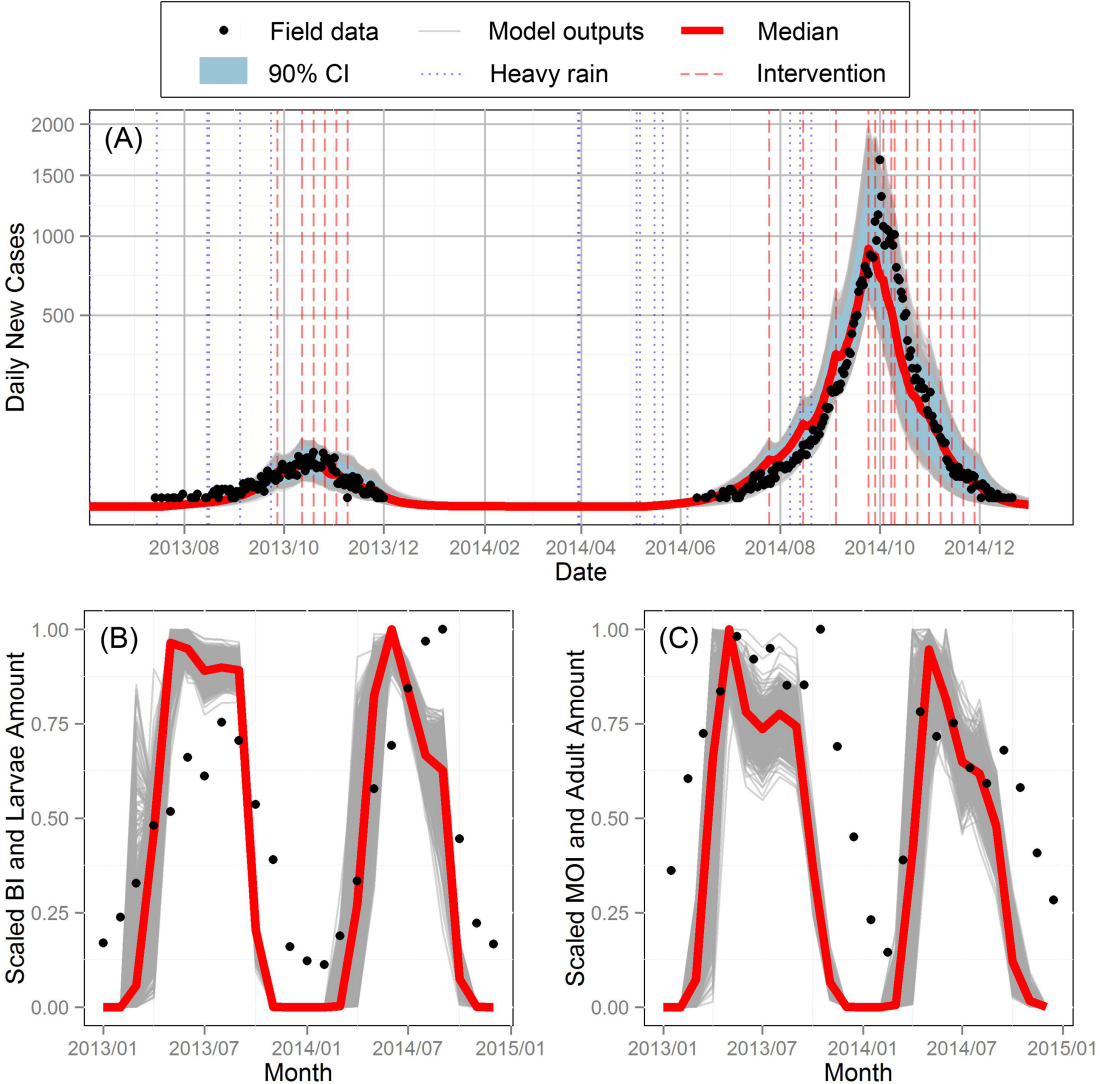
**Model outputs and field data for (A) daily new cases, (B) larvae and (C) adults**. Black dots indicate the number of reported daily new dengue cases in (A), the scaled monthly average BI and MOI in (B) and (C), respectively. Gray and red solid lines represent the model output of each parameter set and the median of these outputs, respectively. Dotted blue and dashed red vertical lines are the dates for heavy rain (part of the immature stages will be washed out if the water level is close to its maximum value) and interventions (insecticide spraying and pooled water removal).

A total of 5 trimming cycles were conducted until the pass and fail univariate distributions offered no further guidance on identifying subspaces with higher pass rates. The results are presented in S1 File. Unlike MCMC fitting, which gives only one parameter set with a confidence interval, RSA gives multiple parameter sets matching the passing criteria. Here we obtained 5,320 parameter sets out of 100,000 runs after the last trimming cycle (Cycle 5) in contrast with the 83 passing sets from 800,000 trials initially (Cycle 1). Fig 3A shows the trajectories and the envelope of the number of daily new cases produced by the 5,320 sets in Cycle 5. As expected, these passing parameter sets produce trajectories which mimic the pattern of the field data successfully, except that the peak number of cases in 2014 is a little lower and occurs earlier than observed. Although the observed initial exponential growth rate of 2014 is higher than that of 2013, the model produces very similar values, presumably because we assume that the intrinsic incubation period, recovery period, transmission probability from vector to human and from human to vector, and reporting rate remain the same from year to year. This lack of fit suggests difference in dengue virus virulence or reporting rate between these 2 years, which may need further investigation in the future.

The number of larvae and adult mosquitoes output by the model shows the same patterns as the mosquito surveillance data for both BI and MOI. Since these entomological datasets were not used in model calibration, they support the validity of our model and its parameterization.

### Stochastic formulation

In transitioning from a purely deterministic model to one suitable for exploring stochastic effects, we must take account of the earlier finding that there are extended regions of the parameter space that led to good fits to the calibration data for the deterministic model. Since thousands of simulations were needed for each parameter set, it is impractical to use all the 5,320 passing sets of Cycle 5. As a result, 100 parameter sets were sampled randomly to represent the space of good fitting vectors (S2 Table). To reduce the computational costs further, a simulation procedure developed specifically for hybrid deterministic/stochastic models was utilized.

The hybrid deterministic/stochastic algorithm can add stochasticity to the dengue transmission model and obtain estimates of the success invasion rate of one imported case and the distribution of FES. This method has been widely used in simulating chemical reactions. It can improve the calculation efficiency significantly while still producing a similar pattern as that achieved by using the exact method [11–13, 32, 33]. The transitions between different compartments here, analogous to the chemical reactions in those studies, were partitioned into “fast” and “slow” subsets according to their transition rates. Fast events happen frequently and have lower level of stochasticity, thus they are simulated by the deterministic model using ordinary differential equations (ODEs) (Events 1–11, 15, 17 and 18 in Fig 2), while slow events happen infrequently so they must be modeled stochastically (Events 12–14, 16, and 19–24). Since there were only small numbers of exposed and infected mosquitoes (*Ae* and *Ai*), and of exposed, infected or recovered humans (*He*, *Hi* and *Hr*) at the beginning of the outbreak, the mortality rates of these compartments (Events 13, 16, 20, 22, and 24) and the transition rates from these compartments (Events 14, 21, and 23) were relatively low. Hence these events are considered as slow events. In addition, the transition rate of Event 12 (bα_hv_*HiAs*/N), which is the mosquito infection via human contagion, is also low, because b is the temperature-dependent biting rate which ranges from 0 to 1; α_hv_ is the transmission probability of dengue virus from infected human to susceptible adult mosquito which also ranges from 0 to 1; *Hi* is the number of infected humans which is a small number at the beginning of the outbreak; and *As*/N is the ratio of mosquitoes to humans, which is also small when compared with mosquito abundance or the human population. Therefore, Events 12 and 19 are slow events, for the same reason.

For the stochastic simulation of the slow events, instead of the widely utilized Gillespie’s stochastic simulation algorithm (SSA) in the chemical studies, here we used the adaptive *tau*-leap algorithm [34–36], which is more commonly used in epidemiological models to reduce the computational burden [10, 34, 35]. To test the validity of the *tau*-leap algorithm, Gillespie’s SSA was also tried for two randomly chosen parameter sets. The Kolmogorov–Smirnov two-sample test for both parameter sets indicated no significant difference between the FES distributions produced by different algorithms. Therefore, only the *tau*-leaping algorithm was applied in the following analysis. The time step *tau* was initially set to be 1/5 day, which is appropriate for a population of the order of millions [37]. If high numbers of events occurred in this time interval which led to negative population sizes, a new value of *tau* was adopted as *tau*/2 to shorten the time interval and avoid negative population sizes. The details of the implementation of the model are shown in S1 File.

### Climate- and intervention-related scenarios

Dengue transmission risk is mainly determined by the mosquito-to-human ratio, temperature, and the immune status of human population [38]. Since the human population and its immune status do not vary much from year to year, the only differences between 2014 and other years were mosquito abundance and temperature. Mosquito abundance further depends on the availability of breeding sites, represented by water level in the model, and human interventions. The most common interventions in Guangzhou are insecticide spraying and emptying water containers, which can reduce the abundance of adults or aquatic stage of mosquitoes immediately. Emptying water containers can also affect the abundance through water availability and the density-dependent rate.

Six different scenarios were designed here to investigate the role of climate and human interventions in determining the potential and FES of the dengue outbreak in 2014 (Table 1). The first scenario used the observed climate data of 2014 and serves as the baseline for comparison (*Scenario 2014*). Then, to study the role of climate only, we replaced the climate files of 2014 in *Scenario 2014* with those of 2013 (*Scenario 2014 to 2013*) and the 30-yr average (*Scenario 2014 to Avg*). The 30-yr average was used here to represent the climate in a typical year. In addition to climate, year 2014 also differed from year 2013 in the initial water level, which was determined by the climate and interventions in the previous year. The initial water level in 2014 is lower than 2013, because more water was removed in 2013 than in 2012 by more frequent interventions. The number of reported dengue cases in Guangzhou increased from 139 in 2012, to 1,249 in 2013, and then to 37,341 in 2014, which led to increasing intervention frequency over these years. Therefore, we compared the results of *Scenario 2013* and *Scenario 2014* to determine the combined effect of climate and intervention. Moreover, aiming to understand the outbreak potential under only natural conditions, we also designed two additional scenarios without interventions (*Scenario 2014 w/o intervention* and *Scenario 2013 w/o intervention*). The previous deterministic model [7] suggested that the interventions can reduce the FES effectively, so here these two scenarios were included to estimate the quantitative effects of intervention in reducing dengue transmission risk.

**Table 1:** Climate file and intervention used in each scenario.

Scenario ID and Name	Climate file	Intervention
(1) *Scenario 2014*	2012 + 2013 + 2014	NA + 2013 + 2014
(2) *Scenario 2014 to 2013*	2012 + 2013 + 2013	NA + 2013 + 2014
(3) *Scenario 2014 to Avg*	2012 + 2013 + 30-yr Avg	NA + 2013 + 2014
(4) *Scenario 2013*	2012 + 2013	NA + 2013
(5) *Scenario 2013 w/o intervention*	2012 + 2013	NA + NA
(6) *Scenario 2014 w/o intervention*	2012 + 2013 + 2014	NA + 2013 + NA

Turning to the role of the timing of imported cases, the introduction date was varied between March 21^st^ and November 26^th^ (the mosquito growth season) at 10-day intervals (a total of 25 different introduction dates). As with the deterministic model, the stochastic model was also run from Jan 1^st^, 2012 to December 31^st^, 2014. Five hundred iterations were run for each combination of scenario and introduction date. The FES of the last simulated year was recorded for each iteration. Therefore, a total of 7.5 million simulations were conducted (100 sample sets, 25 introduction dates, 6 scenarios, and 500 repetitions).

The **successful invasion rate** of an imported case was defined as the proportion of repetitions with a FES greater than 0. The **average final outbreak size** only accounts for successful invasions, and can be calculated as the mean of FESs greater than 0 in 500 repetitions, while the **average final epidemic size** was the mean FES of all invasions.

R 3.2.3 [39] was used for simulation, data analysis and visualization. The package deSolve was used for solving the ODEs [40], Rcpp, foreach, and doSNOW for parallel computing to increase the computational speed [41, 42], and ggplot2 for data visualization [43].

## Results

There are three possible explanations for the early timing of the local transmission in 2014, a higher success rate for a single imported case, more imported cases in early summer, or by chance alone. These possibilities are explored in this section, as well as the distribution and the mean of FES.

### Success rate for a single imported case

**Fig 4:**
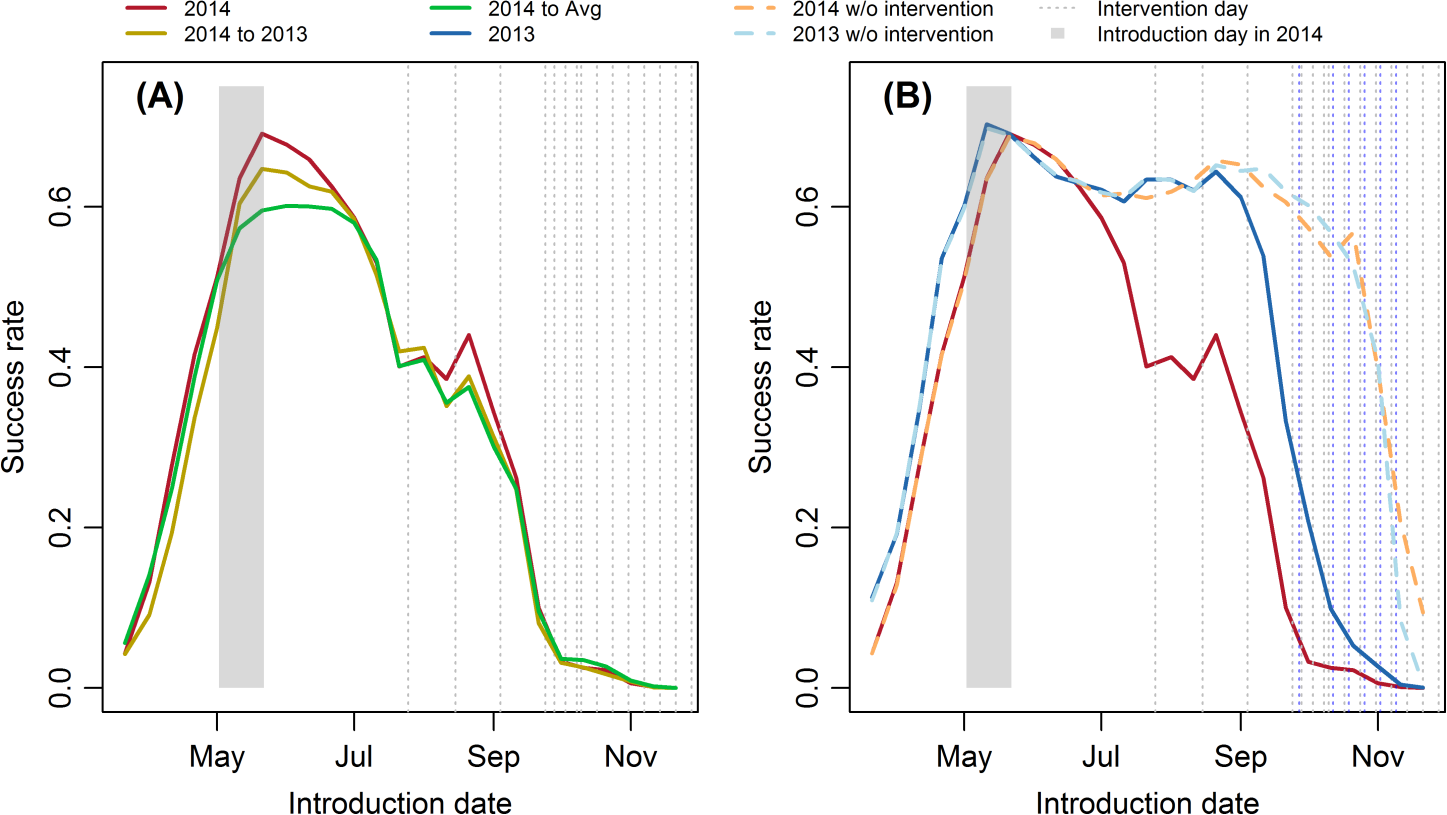
**The mean success rates of 100 parameter sets for (A) *Scenario 2014*, *2014 to 2013* and *2014 to Avg*, and (B) *Scenario 2014*, *2013*, *2014 w/o intervention* and *2013 w/o intervention*.** The shaded rectangle shows the range of the timing of the imported case which started local transmission in 2014 in Cycle 5 of the deterministic model. Blue and gray dashed lines in (B) represent the intervention date in 2013 and 2014, respectively.

In order to determine whether the early outbreak in 2014 was caused by a particularly favorable climate for mosquito growth, we held all the other conditions constant, such as initial water level and the timing of interventions, but only changed the temperature, precipitation and evaporation of 2014 to those of 2013 and then to the 30-yr average (*Scenario 2014 to 2013* and *2014 to Avg*). Fig 4A shows that if an imported case is introduced to the system on the same day in early summer (the shaded rectangle), *Scenario 2014* has the highest success rate. This implies that the climate in 2014 was more favorable for an early outbreak. For all the three scenarios, the success rate increases in late spring and early summer, remains at a high level from May to July, and then decreases until it reaches and stays at a low level in October. The biggest difference in the success rate between these scenarios occurs between May and July, the same period as the peak of success rate.

Fig 4B shows the comparison between two scenarios with observed climate and intervention (*Scenario 2014* and *2013*) and two scenarios with observed climate, but without intervention (*Scenario 2014 w/o intervention* and *2013 w/o intervention*). The result for *Scenario 2014* is depicted here again as a baseline for comparison. Although the success rate of *Scenario 2014* is higher than that of *Scenario 2014 to 2013* and *2014 to Avg* when only the climate is considered (Fig 4A), it is lower than that of *Scenario 2013* when the combined effect of lower initial water level and favorable climate is considered. Therefore, though the climate, especially adequate rainfall, in the early summer of 2014 is more favorable for mosquito growth and dengue transmission, the lower initial water level of 2014 caused by emptying water containers in late 2013 appears to have reduced the risk to a level even lower than that of 2013. In other words, the success rate of a single imported case in causing local transmission was not significantly higher in the time window of interest in 2014 and suggests other explanations, such as more imported cases in that time period or by chance alone.

The difference between *Scenario 2014* and *2014 w/o intervention*, as well as between *Scenario 2013* and *2013 w/o intervention* shows the effectiveness of intervention in reducing dengue transmission probability (Fig 4B). Similar to the three scenarios shown in Fig 4A, the success rates of *Scenario 2013*, *2014 w/o intervention*, and *Scenario 2013 w/o intervention* also increase in the early summer until May. However, the duration of the peak differs. The success rate of *Scenario 2014* stays at the peak until July, *Scenario 2013* until September, while the two scenarios without intervention continue until October. The intervention started on September 27^th^ in 2013 and July 25^th^ in 2014, so they have no influence on the success rate in the beginning of these years. The success rates are the same for *Scenario 2014* and *2014 w/o intervention*, as well as for *Scenario 2013* and *2013 w/o intervention* in the late spring and early summer. However, the success rate drops 20 days before the first intervention, which indicates that the intervention can reduce the transmission probability of a case imported up to 20 days ago. This could possibly due to the death of exposed or infected mosquitoes in the intervention or reduced virus transmission from imported or secondary cases to mosquitoes caused by mosquito population loss.

### Number of imported cases in early summer and outbreak probability

**Fig 5:**
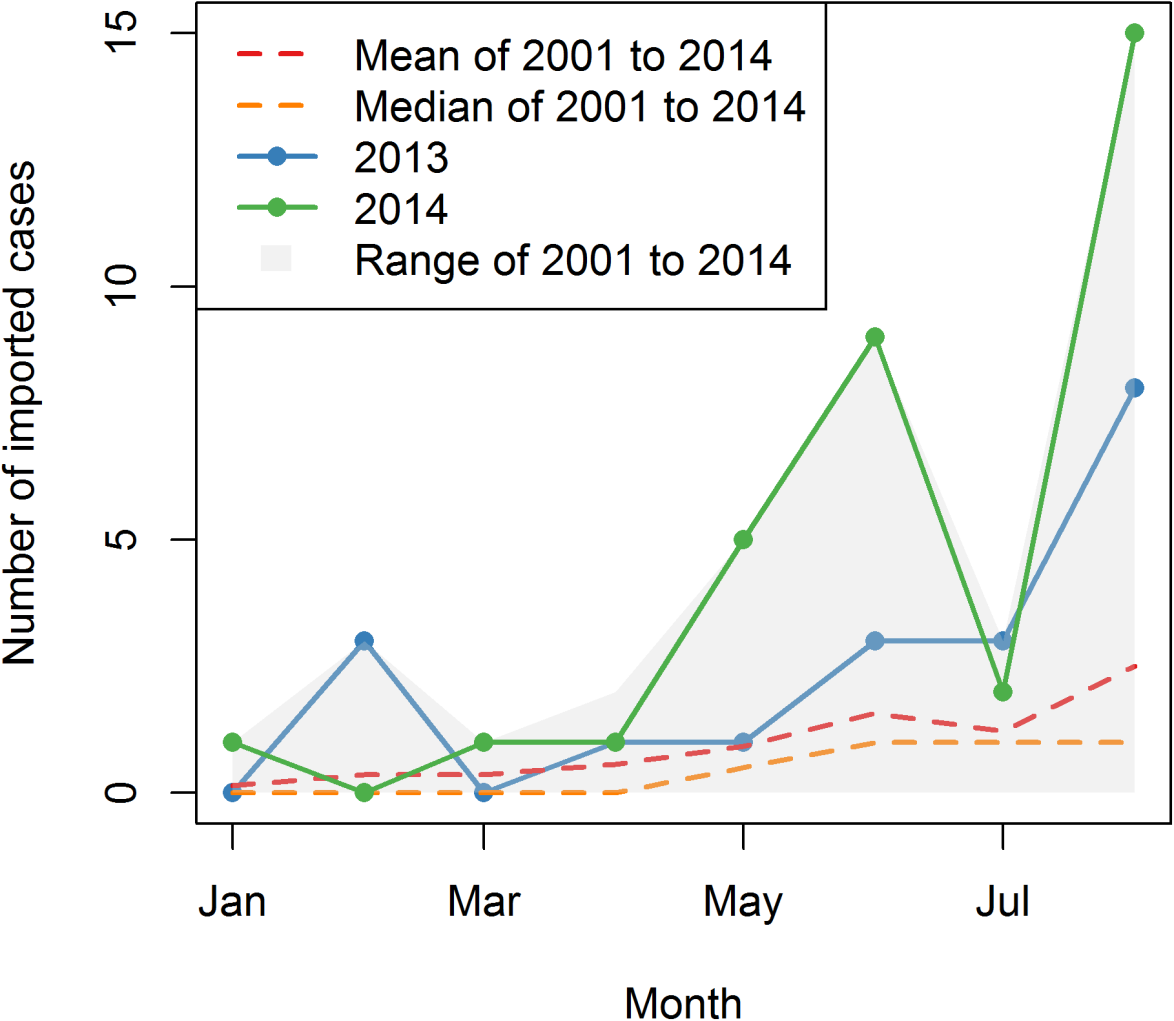
The monthly number of imported cases for 2013, 2014, and the mean, median, and range of 2001 to 2014.

Besides the success rate of one imported case on any given date, the **outbreak probability**, which is the probability that at least one indigenous case occurs in a particular year, also depends on the number of imported cases. It is clear that the outbreak probability will increase when there are more imported cases, although the increase may be quite small depending on the timing pattern. The outbreak probability can be calculated from the success rate of all imported cases in that year, once the timing of each imported case is known. Fig 5 illustrates the number of identified imported cases into Guangzhou from other countries or Chinese cities in 2013, 2014, and the mean, median and range of 2001 to 2014. Since the detection and reporting rate of imported cases are likely to increase after August, 2014, when attention began to be paid to the serious dengue outbreak, only data from January to August are shown here. The number of imported cases known to have entered Guangzhou in May and June, 2014 is four times higher than the average or median of that in 2001 to 2014, which may be the main determinant of the early local dengue virus transmission in 2014.

### Distribution of the final epidemic size

**Fig 6:**
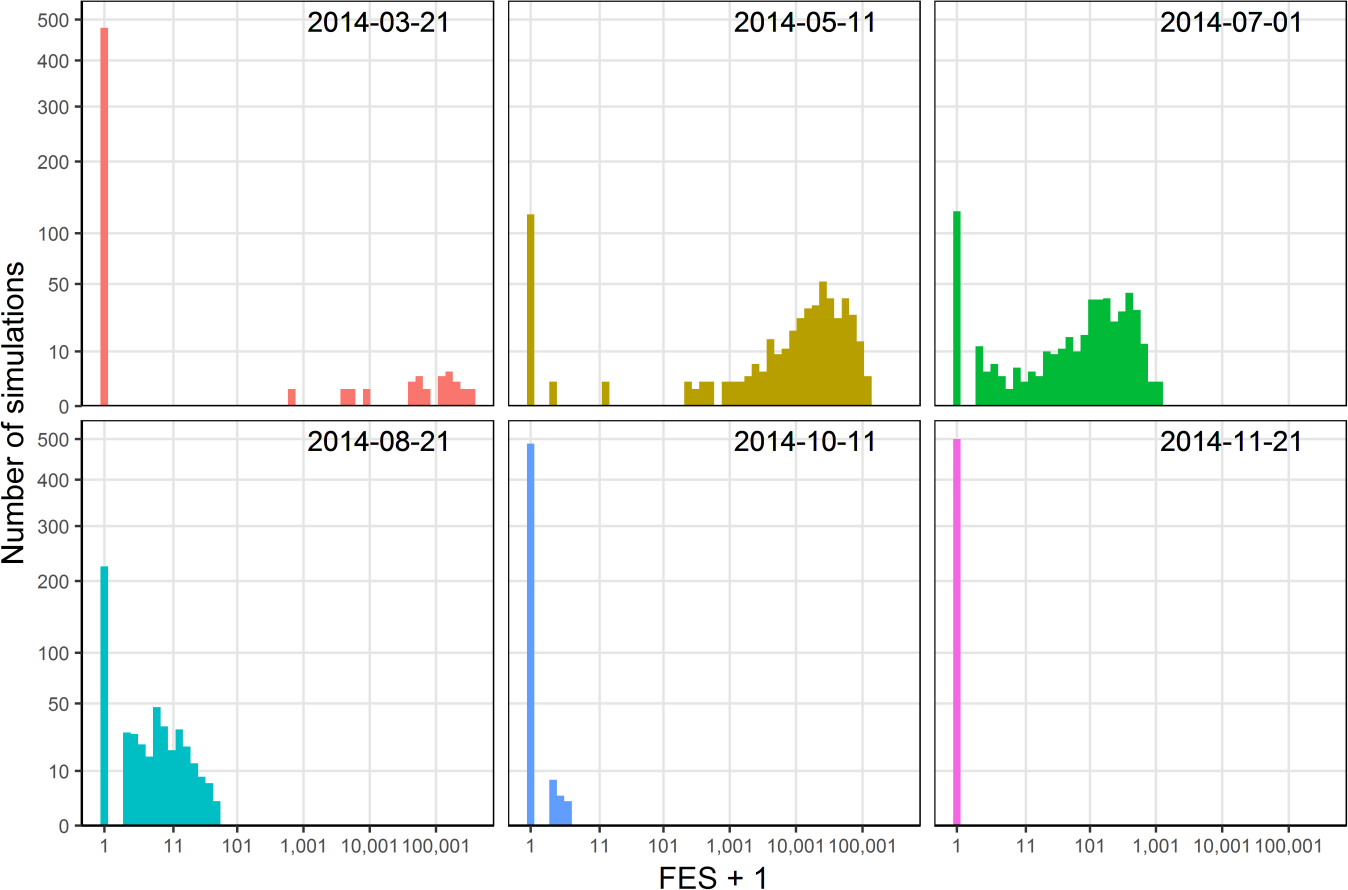
The distribution of FES+1 under Scenario 2014 by using sample set 1.

A different FES can be produced by stochastic effects in each simulation even with the same input. Fig 6 shows the histograms of the FES + 1 under different introduction dates when using the combination of sample set 1 and *Scenario 2014* as an example. The FES for an imported case arriving May 11^th^, 2014, which is around the estimated timing of the first successful invasion in 2014, can range from 0 to 132,457 with a mean of 22,790 and a standard deviation of 25,219. These histograms also show that the FES of successful invasions decreases with the delay of the imported cases. In other words, though the success rate of an early imported case is low, the FES could be extremely high once it succeeds.

### Average final outbreak size and average FES

Average final outbreak size is the mean FES of successful invasions, while average FES is the mean of all simulations. Since the average FES equals the product of the outbreak probability and the final outbreak size, and outbreak probability has been discussed above, here we first look into the average final outbreak size and then the FES. Because all scenarios show the same pattern, and while the results under the last three scenarios are several magnitudes higher than the first three scenarios, to make the figure readable, only the results of the first three scenarios were shown here.

**Fig 7:**
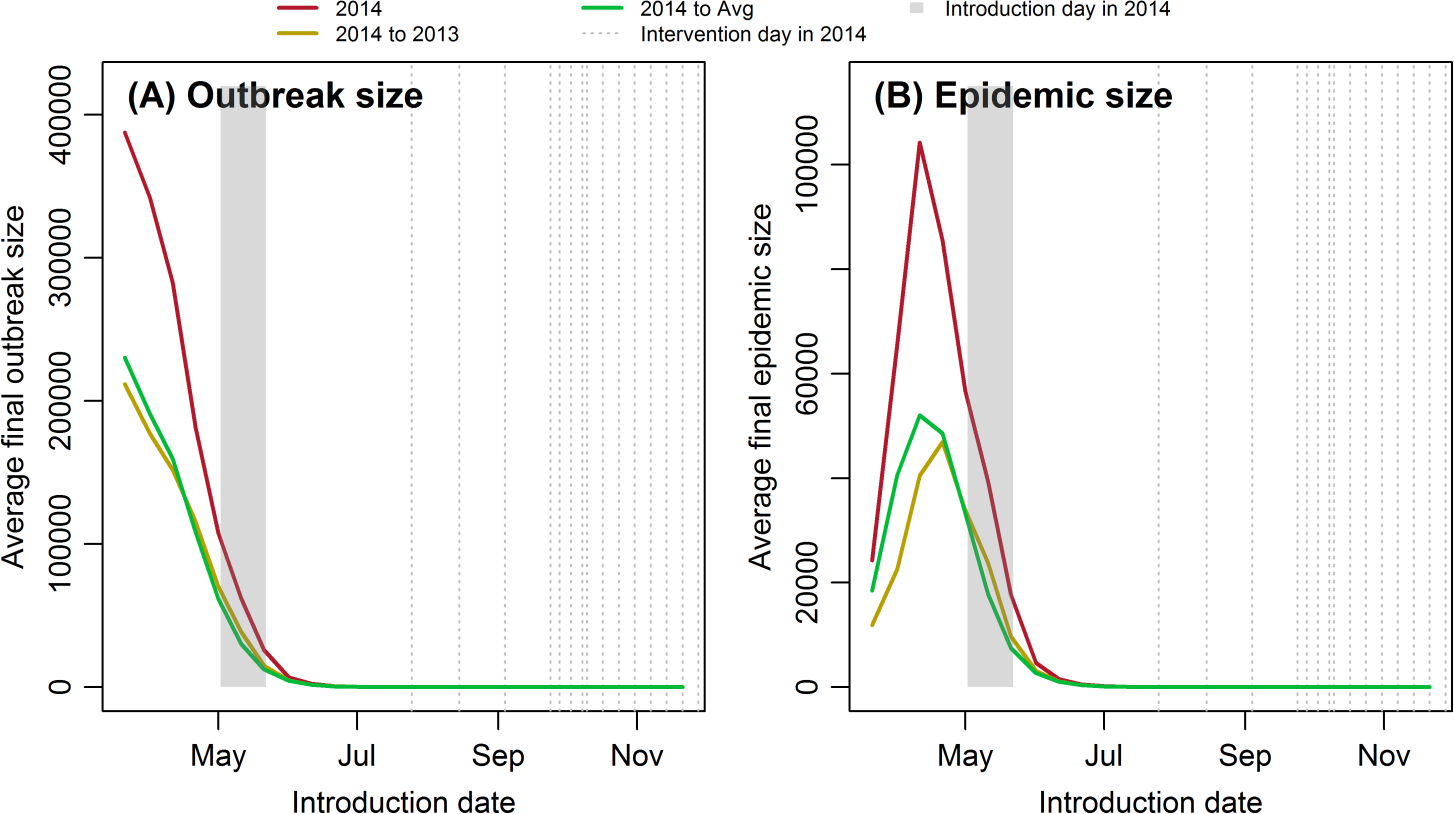
**The (A) average final outbreak size and (B) average FES as a function of introduction date for 100 sample sets.** The shaded rectangle shows the range of the timing of the imported case which started local transmission in 2014 in Cycle 5 of the deterministic model.

Fig 7A shows that the average final outbreak size decreases exponentially with introduction date, and the decrease rate is affected by climate. *Scenario 2014* has the highest average final outbreak size, and *Scenario 2014 to Avg* has the lowest. According to Fig 4A, the mosquito abundance and success rate is higher in *Scenario 2014*, so each case can infect others more easily under this scenario, which will then lead to a higher final outbreak size. Unlike the monotonically decreasing average final outbreak size, the average FES peaks in mid-April (Fig 7B), similar to the conclusion from the deterministic model [7]. In addition, the average FES given by the stochastic model is also comparable to that given by the deterministic model. Though the outbreak size is extremely high before April, the success rate is quite low, while after May, though the success rate reaches its peak, the outbreak size drops dramatically. Therefore, the average FES has the highest value in mid-April, and the peak time changes only slightly with climate. Similar to Fig 4A and Fig 7A, *Scenario 2014* has the highest average FES.

## Discussion

One limitation of the previous deterministic model was that it failed to account for the effect of chance [7]. The transmission dynamics is highly affected by chance at the onset phase of an epidemic, when the populations of infected humans and vectors are low. Under the same situation, an imported case can sometimes result in local transmission, while not at others. However, every imported case can lead to local transmission in the deterministic model when the basic reproduction number R_0_ is greater than one. Therefore, though the deterministic model concluded the early timing of local transmission to be the main determinant of the large FES in 2014, it was unable to answer the question that why it happened earlier in 2014. Here stochasticity was added to the previous model to investigate the successful invasion probability when the importation happens at different times and under different climate and intervention scenarios in 2014.

### Determinants of the early outbreak in 2014

In [9], a stochastic model suggested that the slightly warmer summer was adequate to explain the large dengue outbreak in Madeira, 2012. However, in the Guangzhou outbreak, the favorable climate was only a necessary, but not sufficient condition, because human activities such as intervention and travel also matter. We find the higher number of imported cases in May and June to be the most important determinant of the early outbreak. Although the excessive rainfall in 2014 did increase the successful invasion rate, this effect was cancelled out by the low initial water level due to the interventions in late 2013. Because of a small outbreak in 2013, regular interventions were conducted from late September to early November, which markedly reduced the initial water storage in 2014. The entomological surveillance data shown in Fig 3C supports this interpretation. Specifically, the MOI, which represents the adult mosquito abundance, was lower in 2014 than in 2013, despite the more favorable climate. The increased number of imported cases in late April to early July was also identified as the driving force of the 2014 Guangzhou dengue outbreak by a deterministic model, which incorporated the number of imported case by a fitting function [44]. The result of a classification tree also suggested the number of imported cases, monthly average BI, and temperature as the most important determinants of the dengue outbreak occurrence in Guangzhou from 2002 to 2013 [45].

**Fig 8:**
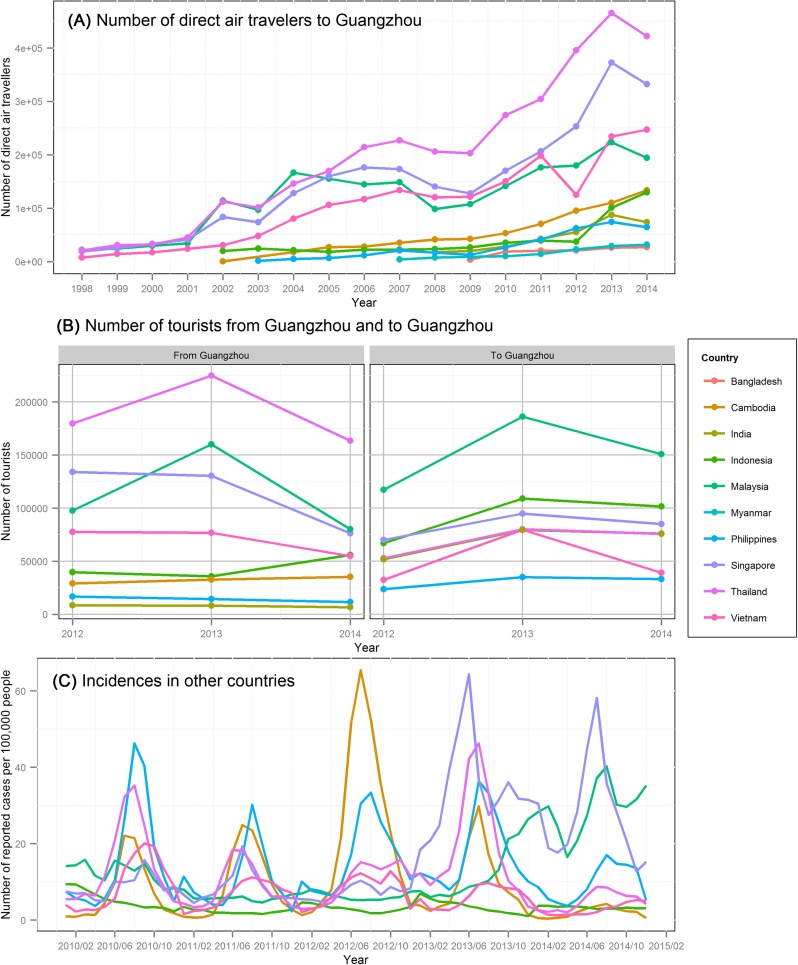
Number of travelers and incidence in surrounding dengue endemic areas. (A) the number of direct air travelers collected from ICAO DATA+; (B) number of tourists from Guangzhou by tour group, and number of foreigners staying overnight in Guangzhou from the Tourism Administration of Guangzhou; (C) reported dengue incidences in the surrounding countries from WHO dengue situation updates and local health departments. Data for Bangladesh and Myanmar is not available for (B) and for Bangladesh, India, and Myanmar is not available for (C).

As noted in Fig 8A and 8B, dengue endemic countries Thailand, Singapore, Malaysia and Vietnam had the highest tourist exchange with Guangzhou. Therefore, more attention should be paid to identifying imported cases from these countries when they have significant dengue outbreaks, especially in late spring and early summer. Though the successful invasion rate is likely to be low in this period, the final outbreak size can be extremely large once it succeeds (Fig 7A). In all countries, the dengue incidences in 2014 were lower than 2013, except for Malaysia (Fig 8C). Although the incidences are somewhat comparable in time, it is questionable to compare the incidence of different countries, because the reporting rates may vary significantly between countries.

A phylogenetic analysis suggested that the dengue virus isolated in Guangzhou, 2014 was most similar to the isolates in Singapore, Malaysia, Indonesia and Thailand [46, 47]. The importation index, which incorporates both the incidence in country of origin and the travel volume between the target city and the country of origin [48, 49], also shows that Singapore, Thailand and Malaysia had the highest importation indices in 2013, and the order changed to Malaysia, Singapore and Thailand in 2014. However, because of the differences in reporting rates between countries, this result is inconclusive. When compared across time, the importation index of 2014 is lower than that of 2013, but more imported cases were observed in 2014, especially in May and June (Fig 5). Considering the epidemic had not started yet at that time, the excessive number of reported imported cases was unlikely to be caused by increasing reporting rate. One possible explanation is spatial heterogeneity. The importation index assumes that human and vector populations are well-mixed, which means that the contact rate between every person and every vector is the same. However, the actual contact rate can be affected by tour route, local transmission hotspots, as well as personal habits, such as whether take measures to avoid mosquito bites. The other explanation is the different reporting rate. For example, if the reporting rate is lower in Malaysia than in other countries, then its actual contribution to the total importation index should be higher, which may make the pattern of the total importation index more similar to that of Malaysia.

### Determinants of success rate

**Fig 9:**
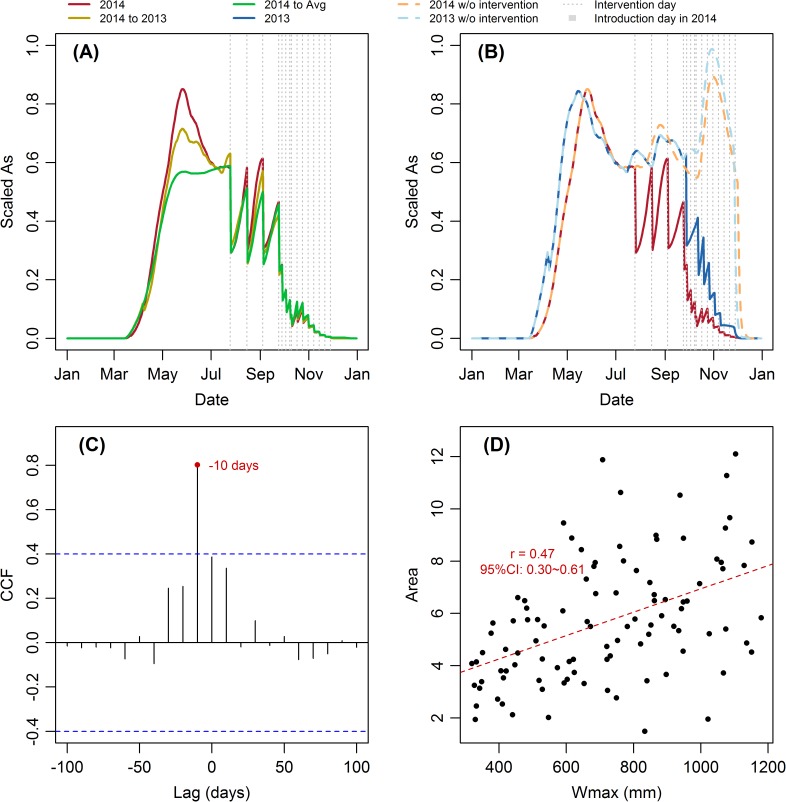
Relationship between *As*, ω_max_ and success rate. Average scaled *As* of 100 parameter sets under (A) *Scenario 2014*, *2014 to 2013*, and *2014 to Avg*, and (B) *Scenario 2014*, *2013*, *2014 w/o intervention*, and *2013 w/o intervention*. Since the absolute values of *As* may vary several magnitude between different parameter sets, the relative values of *As* were used by scaling the As to 0–1 to make them have the same weight on the mean value. (C) The average CCF between the difference of success rate and the difference of *As* under *Scenario 2014*. (D) Scatterplot of ω_max_ in each parameter set and the area between the success rate under Scenario *2014* and *2014 to Avg* of the same parameter set.

The first step of a successful invasion is infecting adult mosquitoes (Event 12), which is described in the model by transition rate bα_hv_*HiAs*/N. Both the biting rate b and the transmission probability from human to vector α_hv_ ranges from 0 to 1, Hi equals to 1, and the total human population N changed little in the study time period. Hence, the size of the susceptible mosquito population, *As*, has the greatest influence on this transition rate. As can be seen from Figs 4, 9A and 9B, it is obvious that the success rate and *As* have almost the same pattern. Furthermore, we calculated the cross-correlation function (CCF) between the difference of success rate and the difference of *As*. First differences were taken here to remove trend of the time series. The result shows that success rate at day t is positively correlated with *As* at day t + 10 for all parameter sets under all scenarios. The average result for *Scenario 2014* is shown in Fig 9C. Since the average recovery period is assumed to be 6 days in the model [7], and we use a 10-day time window for the imported case, the 10-day time lag between *As* and success rate is reasonable.

When we explore the individual results for each of the 100 parameter sets, we found that the gap between the success rate under different climate scenarios is more marked when the maximum water level ω_max_ in that parameter set is larger (Fig 9D). This is because precipitation can affect the carrying capacity through water availability only before ω_max_ is reached, since water will overflow after reach this threshold. Therefore, if the ω_max_ is larger, it takes longer to reach this threshold, which will then lead to larger difference in the mosquito abundance and success rate. This phenomenon implies that reducing ω_max_ by destroying breeding sites can mitigate the effect of climate on dengue transmission dynamics.

### Suggestions for dengue prediction with regression model in subtropical and tropical areas

Regression models are widely used to predict the occurrence of dengue outbreaks, monthly reported cases and the FES [5, 6, 45, 50]. Here we suggest the factors to include in these models according to the results of our stochastic model.

Once given the timing of each imported case, the outbreak probability can be calculated from the success rate curve (Fig 4), which depends mainly on mosquito abundance. Therefore, to predict the occurrence of dengue outbreaks in subtropical areas, variables, such as the number of imported cases and mosquito abundance, should be included. Mosquito abundance is related to temperature, water availability and interventions. Water availability can be estimated from rainfall, evaporation, intervention, and the water limitation parameters ω_max_ and ω_max_. Without all these data, water availability can be indirectly represented by precipitation, and relative humidity. However, precipitation and relative humidity do not reflect the effects of intervention and the relationship between water level and precipitation or relative humidity is non-linear. The water level increases with precipitation in spring and early summer, but when it reaches its threshold ω_max_, the water level cannot increase further and remains at this value until evaporation outweighs precipitation or the occurrence of an intervention. In addition, the relationship between water level and mosquito abundance is complicated, which depends on previous water level, the environmental carrying capacity and the previous abundance of aquatic stages. As a result, mosquito indices might still be preferred among all these variables. By including the monthly maximum and minimum temperature, average BI, and number of imported cases, a classification tree can be used to predict the dengue outbreak occurrence in Guangzhou [45]. For tropical areas, since local epidemics continue throughout the entire year, there is no need to predict the outbreak occurrence.

The FES depends on both the final outbreak size and outbreak probability. The final outbreak size decreases exponentially as a function of the introduction date, while temperature, water storage, and human interventions can modify the decrease rate through changing the force of infection. Earlier introduction in subtropical areas means a longer period of transmission and more cases, because dengue transmission is terminated by the low temperature every winter. The force of infection can be affected by mosquito abundance, biting rate and virus virulence. Although both the length of transmission and the force of infection can affect the final outbreak size exponentially, the former fluctuates over a much wider range. Sometimes, though the climate is favorable for vector growth, no big outbreak occurs because of the low level or late importation. Thus the imported cases introduce high uncertainty to the prediction models, and a successful model should ideally include the number of imported cases and indigenous cases in the previous month to reduce this uncertainty. Time series regression models with the number of imported and indigenous cases in the previous month, lagged temperature, precipitation and relative humidity can give reasonable estimations of the number of new cases [6, 50]. Climate conditions in tropical areas can support overwinter local transmission, so the role of imported cases here is not as important as that in subtropical areas. Climate and interventions are the only crucial factors in tropical areas. Prediction models based on only climate can have good prediction power [51–53]. When considering the spatial aspects of the transmission, heterogeneity in the distribution of population, open water, vegetation, and host immunity can also influence the outbreak probability and FES for both subtropical and tropical countries [54, 55].

### Implications for intervention

Currently, the most common interventions in China are chemical insecticide spraying and environmental management, such as water container emptying. Also, releasing mosquito larvae-eating fish *Gambusia affins* and *wolbachia*-infected male mosquitoes are sometimes used in small scale tests.

Since the adult abundance and success invasion rate of imported cases increase sharply in April and May (Fig 4), the interventions should begin before or in this time period. However, the regular interventions in Guangzhou started on September 27^th^ and July 25^th^ in 2013 and 2014, respectively, both of which were at least one month later than the onset of local epidemics. In addition, interventions out of the transmission season, such as before the next transmission season can also be important, because it can reduce the water storage, increase the time needed to accumulate water in the next transmission season, and postpone the outbreak, which, in turn, can then decrease the final outbreak size significantly.

According to result of the PRECIS (Providing Regional Climate for Impact Study) regional climate modeling system, the spring and summer rainfall of South China will increase in the future [56], which may increase the average FES by causing earlier outbreaks. However, destroying mosquito breeding sites can mitigate this detrimental impact of climate change, since it can reduce the maximum water level ω_max_ and narrow the gaps in the success rate or final outbreak size between different climate scenarios (Fig 9D).

Increasing international travel, urbanization, global warming, and changes in precipitation patterns pose higher dengue outbreak risk for Guangzhou and other subtropical non-endemic areas. Moreover, secondary infection can worsen the situation by increasing the incidence of life-threatening dengue hemorrhagic fever and shock syndrome [57]. Under this situation, vector control and management of imported cases at entry points should be implemented strictly, especially around mid-April, to reduce possible health and economic loss caused by dengue or other mosquito-borne diseases, such as yellow fever and Zika. Since the travel volumes between Thailand, Malaysia, Singapore and Guangzhou are the highest, more attentions should be paid to the detection of imported cases when dengue outbreaks occur in these countries, especially at early summer. Besides international imported cases, cases from other Chinese cities should also be considered. Since Guangzhou is normally the first city to have dengue outbreak sin mainland China, it is unusual for Guangzhou to have domestic imported cases in the early phase of an epidemic, although it happens on occasion. For example, the epidemic in Zhongshan started 2 weeks earlier than in Guangzhou [50]. On the other hand, the onset of an epidemic in Guangzhou signals that surrounding cities of the need to pay attention to case detection and vector control immediately. For example, from Figs 4B and 9C, it can be seen that, after detection, the model suggests that interventions should be conducted around the residences of the reported cases whose symptoms began less than 10 days ago, even if they have recovered.

### Limitations

The model proposed here represents the transmission of only one serotype DENV-1, since 5,947 out of 6,024 cases (98.7%) tested in 2014 were infected by DENV-1, and only 74 and 3 were infected by DENV-2 and -3, respectively [48]. When adapted for use in other subtropical areas, the single serotype assumption may or may not be appropriate. Secondly, we assumed that dengue virus virulence and reporting rate were the same for 2013 and 2014, which led to similar initial exponential growth rates for these two years. However, from the daily reported new case data, the growth rate of 2014 was slightly higher than that of 2013 (Fig 3), the reasons for which deserve further investigation. Thirdly, the estimated success rates illustrated in Fig 4 are estimates of the upper bound on what might be expected in practice, because it estimates the success rates for imported cases who spend the whole viremic period in Guangzhou. However, an imported case can enter Guangzhou at the middle or even the last day of the viremic period and thereby have less time to infect *Ae*. *albopictus* and result in a lower successful invasion rate. In this model, we also assumed that humans and the mosquitoes are well-mixed, which means the contact rate is the same for every host and vector. However, studies of *Ae*. *albopictus* indicate that their flying range is as small as 300 meters, and that they normally they stay near their breeding sites for their whole life [58]. Therefore, a spatially-explicit model is needed to better simulate the spatial distribution of dengue cases and study the risk factors related to the observed distribution patterns in the future.

## Supporting information

S1 File Model details. (DOCX)

S1 Table Number of foreign tourists staying overnight in Guangzhou and number of tourists traveled from Guangzhou with tour groups. (XLSX)

S2 Table The 100 parameter sets used in the sthochastic model. (XLSX)
