# Supplementary material for: The interplay of climate, intervention and imported cases as determinants of the 2014 dengue outbreak in Guangzhou
Source: PLoS Negl Trop Dis. 2017 Jun 22;11(6):e0005701. doi: 10.1371/journal.pntd.0005701 (PMC5507464; doi:10.1371/journal.pntd.0005701)
Supplement: S1 File — (DOCX) [file pntd.0005701.s001.docx]

S1 File. Model details

# State variables and initial values

The initial value for E is not important since it can only affect the mosquito abundance of the first year. In other words, the same stable population will be reached in 2013 no matter what the initial value was in 2012. The initial value for L is set to be 1 to avoid division by zero when calculating Aeu. The initial value of Hs was set to be the population in Guangzhou at the end of 2011.

Table S1. State variables and initial values in the model

| State Variable | Definition | Initial value |
| --- | --- | --- |
| E | Eggs | 100,000 |
| L | Larva | 1 |
| P | Pupa | 0 |
| Aeu | Emerging mosquito adults | 0 |
| As | Susceptible mosquito adults | 0 |
| Ae | Exposed mosquito adults | 0 |
| Ai | Infectious mosquito adults | 0 |
| Hs | Susceptible human | 12,700,799 |
| He | Exposed human | 0 |
| Hi | Infectious human | 0 |
| Hr | Recovered human | 0 |

# Ordinary differential equations (ODEs), events, parameters and functions in deterministic model

## 2.1 ODEs

Only females were considered in the adult stage by times the sex ratio ξ when calculating Aeu.

$$\frac{\mathrm{dE}}{\mathrm{dt}}=n_{e}f_{ag}\left( As+Ae+Ai \right)-{\kappa f}_{E}E-\mu_{E}E$$

$$\frac{\mathrm{dL}}{\mathrm{dt}}=\kappa f_{E}E-f_{L}L-m_{L}L$$

$$\frac{\mathrm{dP}}{\mathrm{dt}}=f_{L}L-f_{P}P-m_{P}P$$

$$\frac{\mathrm{dAeu}}{\mathrm{dt}}={\xi e^{-\mu_{em}(1+\frac{P}{LReal})}f}_{P}P-1/\gamma_{aem}Aeu-m_{A}Aeu$$

$$\frac{\mathrm{dAs}}{\mathrm{dt}}=1/\gamma_{aem}Aeu-b\alpha_{hv}\frac{Hi}{N}As-m_{A}As$$

$$\frac{\mathrm{dAe}}{\mathrm{dt}}={b\alpha_{hv}\frac{Hi}{N}As-f}_{exv}Ae-m_{A}Ae$$

$$\frac{\mathrm{dAi}}{\mathrm{dt}}=f_{exv}Ae-{\sigma m}_{A}Ai$$

$$\frac{\mathrm{dHs}}{\mathrm{dt}}=\alpha_{H}N-b\alpha_{vh}\frac{Ai}{N}Hs-\mu_{H}Hs$$

$$\frac{\mathrm{dHe}}{\mathrm{dt}}=b\alpha_{vh}\frac{Ai}{N}Hs-{{1/\tau}_{exh}He-\mu}_{H}He$$

$$\frac{\mathrm{dHi}}{\mathrm{dt}}={1/\tau_{exh}He-{1/\tau}_{ih}Hi-\mu}_{H}Hi$$

$$\frac{\mathrm{dHr}}{\mathrm{dt}}={{1/\tau}_{ih}Hi-\mu}_{H}Hr$$

$$N=Hs+He+Hi+Hr$$

## 2.2 Water level

The water level (ω_Real_) in the system was calculated as:

$$\omega_{Real+1}=\left\{ c*\begin{matrix} {c*\omega}_{max} - EV, & \omega_{Real} + RF - EV > \omega_{max} \\ \omega_{Real}+RF - EV, & \omega_{min}< \omega_{Real}+RF - EV < \omega_{max} \\ c*\omega_{Real}+RF, & \omega_{Real} + RF - EV < \omega_{min} \end{matrix} \right. (1)$$

These equations are similar to those in [2], but here we included the water removed by the regular interventions. The coefficient c equals μ_i_ when intervention takes place on that day and 1 otherwise, which means a fraction (1-μ_i_) of the water is removed by emptying water containers, and only μ_i_ of the water is left in the system and continues to serve as potential mosquito breeding sites. μ_i_ is a parameter needed to be estimated from the deterministic model (Table S3).

## 2.3 Events in the deterministic model

The events in this part is different from the events in the stochastic model. Here, the events lead to sudden changes in the state variable, which will be ignored by the integration method if they are simply included by the derivation function. These events are organized as a data frame, and then input to the solver contained in the deSolve package. [[1](#_ENREF_1)]

**Spillover effect [**[**2**](#_ENREF_2)**]**

When the water level is close to its maximum value ω_max_ and a heavy rain (> 50 mm in 24 hours) occurs, a fraction ω of the aquatic stage mosquitos (Egg, larvae, pupae) will be washed out from the water containers. The fraction ω is calculated as:

$$\omega=\omega_{0}\times\frac{1.2{\times(\frac{\omega_{Real}}{\omega_{max}})}^{20}}{1+1.2{\times(\frac{\omega_{Real}}{\omega_{max}})}^{20}}$$

**Imported cases**

Since the temperature in winter is too low to support the overwinter transmission of dengue virus, introduction of the virus by imported cases is required every year to start the local transmission. In the deterministic model, one case alone is enough to start the epidemic. But since it is impossible to know the exact timing of the imported case which starts the successful local transmission, due to the asymptomatic and unreported case, the timing of the imported cases in 2013 and 2014 are treated as two parameters β_2013_ and β_2014_. On these days, we add 1 to the Hi to represent the introduction of imported case.

**Intervention**

Interventions, such as adulticide spraying and pooled water removal, were conducted regularly on every Friday afternoon from October 9^th^ to November 9^th^ in 2013, and from September 24^th^ to November 28^th^, as well as on July 25^th^, August 15^th^, and September 4^th^. We assumed that only a fraction μ_a_ of the adults survive the spraying, and only μ_i_ of the water and immature stages still stay in the water container.

## 2.4 Constant parameters

Table S2. Constant parameters in the model

| Parameter | Definition | Typical value | Note |
| --- | --- | --- | --- |
| κ | Binary variable. 0 for diapausing period and 1 otherwise. | 0 or 1 | 0 from Oct 25^th^ to Mar 15^th^, 1 otherwise [[3](#_ENREF_3)] |
| μ_H_ | Human morality rate in Guangzhou | 0.000035 | 1/Average life expectancy estimated from [[4-6](#_ENREF_4)] |
| α_H_ | Human birth rate in Guangzhou | 0.000081 | Estimated from [[4-6](#_ENREF_4)] |
| ξ | Sex ratio of Ae. albopictus | 0.5 | From experiments in [[7](#_ENREF_7),[8](#_ENREF_8)] |

## 2.5 Parameters need to be estimated

The range for the timing of imported case is calculated as the timing of local transmission – 15 days ± 25 days. Fifteen days represent the sum of typical extrinsic and intrinsic incubation period in summer. For example, the local transmission in 2013 started on Day 561, then the range for β_2013_ is calculated as (561 – 15 - 25, 561 – 15 + 25), which is Day 521 to Day 571.

Table S3. Parameters need to be estimated in the deterministic model

| Parameter | Definition | Typical values | Reference |
| --- | --- | --- | --- |
| μ_E_ | Mortality rate of eggs (day^-1^) | 0 – 0.1 | [[9](#_ENREF_9)] |
| θ | The ratio of minimum to ideal egg hatching rate | 0 – 1 | To our best knowledge |
| λ | The ratio of minimum to ideal larval development rate | 0 – 1 | To our best knowledge |
| ω_0_ | The maximum washout fraction by heavy rain | 0 – 1 | To our best knowledge |
| ω_min_ | Lowest water level in the system, representing water in shaded area, containers with lids, or other water shielded from evaporation (mm) | 0 – ω_max_ | [[2](#_ENREF_2)] |
| ω_max_ | Highest water level in the system, beyond which overflow will occur (mm) | 200 – 2000 | [[2](#_ENREF_2)] |
| π_max_ | The maximum carrying capacity for the aquatic stages (eggs, larva, and pupa) | 1.0 * 10^6^ – 1.2 * 10^7^ | To our best knowledge |
| γ_aem_ | Duration of the emergence (day) | 1 – 7 | [[8](#_ENREF_8),[10](#_ENREF_10),[11](#_ENREF_11)] |
| μ_em_ | Mortality rate during the emergency (day^-1^) | 0 – 0.2 | [[9](#_ENREF_9)] |
| σ | The ratio of infected to uninfected mortality of Ae. albopictus for both immature and adult phase | 1 – 3 | [[12](#_ENREF_12)] |
| τ_exh_ | Intrinsic incubation period (day) | 3 – 9 | [[13](#_ENREF_13)] |
| τ_ih_ | Recovery time (day) | 3 – 9 | [[14](#_ENREF_14)] |
| α_vh_ | Transmission probability of dengue virus from infected vector to human | 0 – 1 | To our best knowledge |
| α_hv_ | Transmission probability of dengue virus from infected human to vector | 0 – 1 | To our best knowledge |
| φ | Reporting rate | 0 – 1 | To our best knowledge |
| β_2013_ | Timing for the imported case in 2013 (Jan 1^st^, 2012 as Day 1) | 521 – 571 (Jan 1^st^, 2012 as Day 1) | Outbreak started on Day 561 |
| β_2014_ | Timing for the imported case in 2014 (Jan 1^st^, 2012 as Day 1) | 853 – 903 | Outbreak started on Day 893 |
| μ_a_ | Survival rate of adult mosquitos in adulticide spraying | 0 – 1 | To our best knowledge |
| μ_i_ | Survival rate of the immature mosquitos in pooled water removal | 0 – 1 | To our best knowledge |

## 2.6 Temperature-dependent rates

The mortality of pupa and adults, the development rate from pupae to emerging adult, duration of gonotrophic cycle and EIP, biting rate, and eggs laid per gonotrophic cycle all depend on temperature. In addition, the ideal development rates of eggs and larva, and the ideal mortality rate of larva also depend on temperature. Then the ideal rates and water level are used to calculate the real rates under current density.

The form of the development rates of eggs, larva, and pupa, the gonotrophic cycle duration and EIP is based on enzyme kinetics model in [[15](#_ENREF_15)]. And the coefficients in the equation are estimated from experiments conducted in Guangzhou and surrounding areas. [[11](#_ENREF_11),[16](#_ENREF_16)] The form of these rates is as follows:

$$r\left( T_{t} \right)=\frac{\rho(25℃)\times(T_{t}/298){\times e}^{\frac{\Delta H_{A}}{R}(\frac{1}{298}-\frac{1}{T_{t}})}}{1+e^{\frac{\Delta H_{H}}{R}(\frac{1}{T_{1/2H}}-\frac{1}{T_{t}})}}$$

Here, T_t_ is the average temperature (°K) on day t; r(T_t_) is the development rate (hr^-1^) at T_t_; ∆H_A_ and ∆H_H_ are the enthalpy of the activation of reaction that catalyzed by the enzyme (cal mol^-1^) and the enthalpy change associated with high temperature inactivation of the enzyme (cal mol^-1^), respectively; T_1/2H_ is the temperature at which half of the enzyme is inactived because of high temperature; and R is the universal gas constant (1.987 cal mol^-1^ deg^-1^). Then the coefficient ρ(25℃), ∆H_A_, ∆H_H_, and H_1/2H_ were estimated.

The idea mortality rate of larva, mortality rate of pupa and adults, biting rate and the eggs per gonotrophic cycle were estimated by using quadratic or piecewise functions [9,10].

| Table S4. Temperature-dependent rates | | |
| --- | --- | --- |
| Function | Definition | Expression |
| feideal | Ideal development rate of eggs (day^-1^) | $feideal= 24\times\frac{0.00835\times(T_{t}/298){\times e}^{\frac{46701.2}{R}(\frac{1}{298}-\frac{1}{T_{t}})}}{1+e^{\frac{309796.0}{R}(\frac{1}{313.511}-\frac{1}{T_{t}})}}$ |
| mlideal | Ideal mortality rate for of (day^-1^) | $mlideal= \left\{ \begin{matrix} 0.0000866T^{2}-0.00368T+0.0451, & T\geq12.5℃ \\ 0.5, & T<12.5 ℃ \end{matrix} \right.$ |
| flideal | Ideal development rate of larva (day^-1^) | $flideal= 24\times\frac{0.00608\times(T_{t}/298){\times e}^{\frac{51681.3}{R}(\frac{1}{298}-\frac{1}{T_{t}})}}{1+e^{\frac{186888.0}{R}(\frac{1}{313.208}-\frac{1}{T_{t}})}}$ |
| mpideal | Mortality rate of pupa (day^-1^) | $mp= \left\{ \begin{matrix} 0.01, & 12.5℃\leq T\leq35.0℃ \\ 0.5, & else \end{matrix} \right.$ |
| fp | Development rate of pupae to emerging adults (day^-1^) | $fp= 24\times\frac{0.0143\times(T_{t}/298){\times e}^{\frac{44093.2}{R}(\frac{1}{298}-\frac{1}{T_{t}})}}{1+e^{\frac{100261}{R}(\frac{1}{330.058}-\frac{1}{T_{t}})}}$ |
| ma | Mortality rate of adults (day^-1^) | $ma= \left\{ \begin{matrix} 0.000114T^{2}-0.00427T+0.0639, & T\geq15.0℃ \\ 0.5, & T<15.0 ℃ \end{matrix} \right.$ |
| fag | 1/Duration for gonotrophic cycle (day^-1^) | $fag= 24\times\frac{0.0102\times(T_{t}/298){\times e}^{\frac{60513.2}{R}(\frac{1}{298}-\frac{1}{T_{t}})}}{1+e^{\frac{705550}{R}(\frac{1}{308.352}-\frac{1}{T_{t}})}}$ |
| fexv | 1/Extrinsic incubation period (day^-1^) | $fexv= 24\times\frac{0.00333\times(T_{t}/298){\times e}^{\frac{70802.6}{R}(\frac{1}{298}-\frac{1}{T_{t}})}}{1+e^{\frac{177239}{R}(\frac{1}{448.619}-\frac{1}{T_{t}})}}$ |
| b | Biting rate (day^-1^) | Max(-0.004981T^2^+0.274T -2.94,0) |
| n_e_ | Eggs per gonotrophic cycle (per female) | Max(-0.5717T^2^+31.8313T-349.8819,0) |

## 2.7 Density-dependent rates [[2](#_ENREF_2)]

The real development rate of egg and larva, and the real mortality rate of larva depend not only on temperature, but also on density, or water level in other words. First the current water level is calculated according to precipitation, evaporation, ω_min_ and ω_max_, then the real carrying capacity L_Real_ under current water level is calculated as a proportion ω_real_ /ω_max_ of π_max_. Then the ideal rate, current water level and real carrying capacity rate are used to calculated the density-dependent rates.

Table S5. Density-dependent rates

| Function | Definition | Expression |
| --- | --- | --- |
| L_Real_ | The carrying capacity of mosquito larvae population | L_Real_ =π_max_ *$\frac{\omega_{real}}{\omega_{max}}$ |
| fe | Real egg development rate (day^-1^) | $fe=\left( feideal-feideal*\theta\right)*\frac{20\left( \frac{\omega_{real}}{\omega_{max}} \right)^{8}}{1+20\left( \frac{\omega_{real}}{\omega_{max}} \right)^{8}}+feideal*\theta$ |
| fl | Real larva development rate (day^-1^) | $fl=\left( flideal-flideal*\lambda\right)*\frac{2\left( \frac{L}{L_{Real}} \right)^{-1}}{1+2\left( \frac{L}{L_{Real}} \right)^{-1}}+flideal*\lambda$ |
| ml | Real mortality for larva (day^-1^) | ml = mlideal*(1+L/L_Real_) |

# Calibration of the deterministic model

The deterministic model was calibrated by a strategy named regional sensitivity analysis (RSA). [[17](#_ENREF_17)] The details for the calibration can be found in [[18](#_ENREF_18)]. The randomly sampled parameter sets were used to run the model and kept in 2 sets according to whether the result meet all the 8 criteria listed below, which are also illustrated in Fig S1:

(1) The number of daily new cases of at least one day in the time window between August 22^nd^ and September 11^th^, 2013 is greater than 0 and lower than 10;

(2) The daily new cases peaked between October 9^th^ and October 29^th^ in 2013;

(3) The peak amount of daily new cases in 2013 is greater than 10 and lower than 60;

(4) The number of daily new cases of at least one day in the time window between November 20^th^ and December 10^th^, 2013 is greater than 0 and lower than 10;

(5) The number of daily new cases of at least one day in the time window between August 2^nd^ and August 12^th^, 2014 is greater than 5 and lower than 60;

(6) The daily new cases peaked between September 21^st^ and October 11^th^ in 2014

(7) The peak amount of daily new cases in 2014 is greater than 600 and lower than 2000;

(8) The number of daily new cases of at least one day in the time window between November 10^th^ and November 20^th^, 2014 is greater than 5 and lower than 60.

The number of daily cases output by the model is calculated as He*τ_exh_*φ.


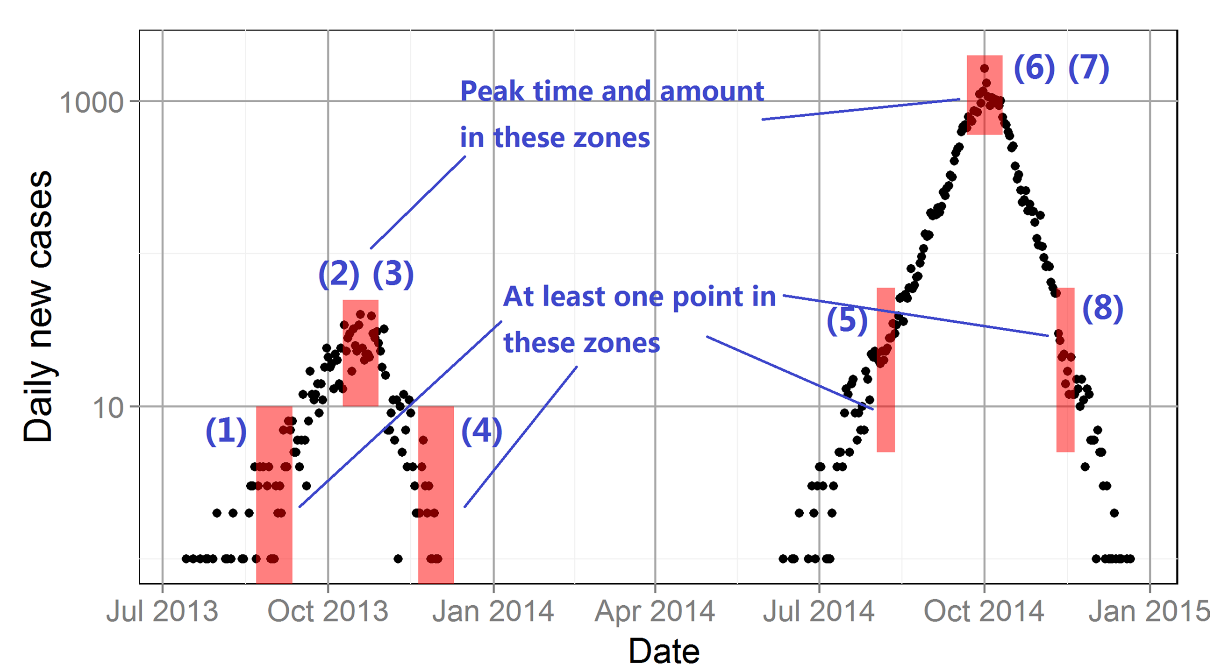


Fig S1. The daily reported new cases and the 8 passing criteria for the deterministic model. Black dots represent the number of daily new cases reported to Guangzhou CDC, and the red shaded rectangles show the time and amount window for the 8 criteria. Source: [[18](#_ENREF_18)]

Then the range of each parameter was trimmed according to the Kolmogorov plot, which shows the difference of the cumulative distribution function (CDF) between pass and fail group. The high or low end of the range which contains only few passing values was removed in the next running cycle. By doing this, we narrowed down the parameter space and tried to find a smaller space with higher passing rate. The passing rate, Kolmogorov plot, and test result for the 5 cycles are shown in the following figures and tables.

**Cycle 1** using a wide range for each parameter

Passing rate: 83/800,000 = 0.0104%


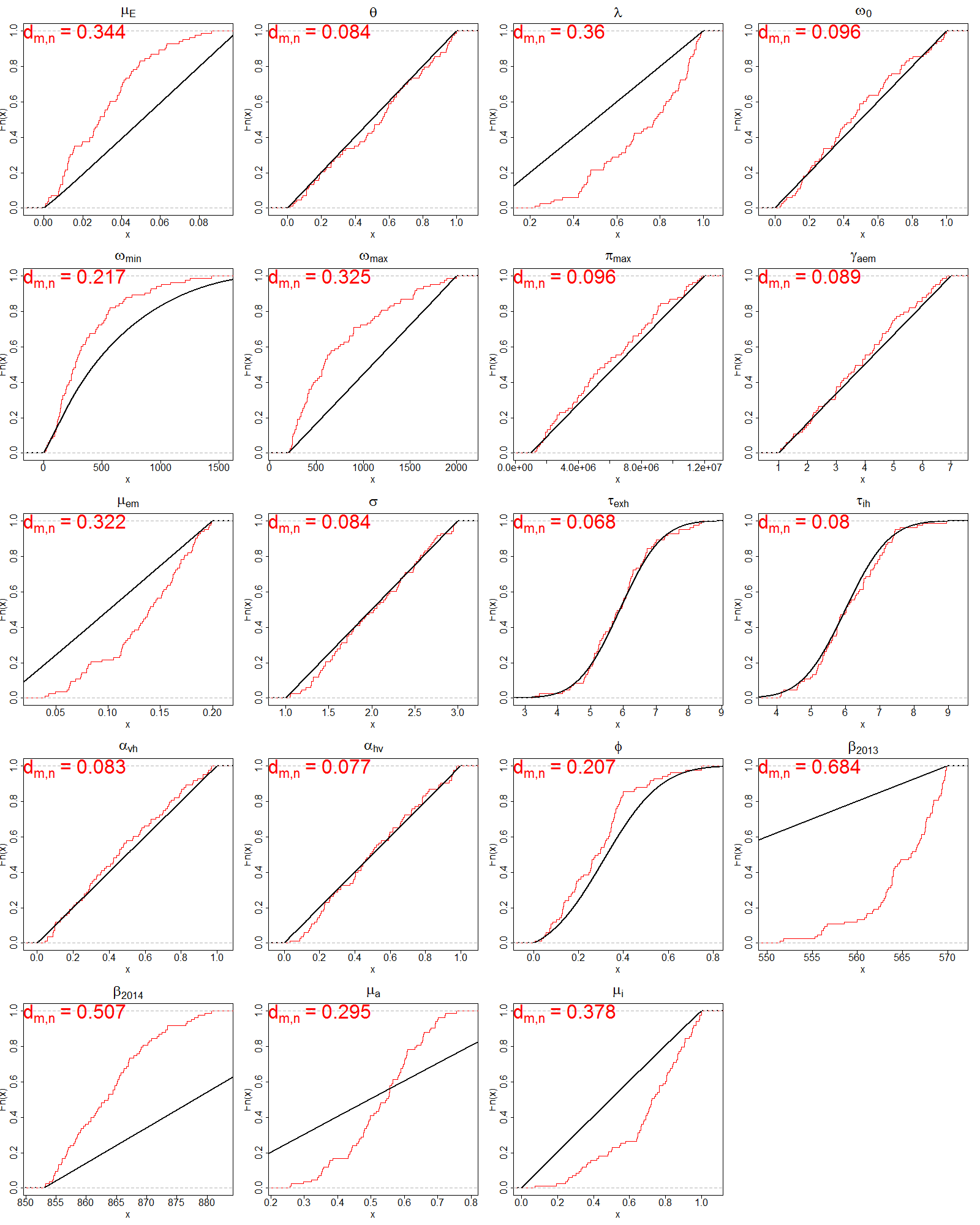


Fig S2. The CDF for pass (red) and fail (black) groups in Cycle 1

Table S6. The mean and standard deviation of pass and fail group, and the value d_m,n_ and the p-value of Kolmogorov – Smirnov statistic in Cycle 1

|  | PassMean | FailMean | PassStd | FailStd | d_m,n_ | p-value |
| --- | --- | --- | --- | --- | --- | --- |
| μ_E_ | 0.031 | 0.051 | 0.021 | 0.029 | 0.344 | 0.000 |
| θ | 0.525 | 0.499 | 0.292 | 0.289 | 0.084 | 0.607 |
| λ | 0.725 | 0.500 | 0.211 | 0.289 | 0.360 | 0.000 |
| ω_0_ | 0.472 | 0.499 | 0.279 | 0.289 | 0.096 | 0.423 |
| ω_min_ | 367 | 550 | 312 | 437 | 0.217 | 0.001 |
| ω_max_ | 756 | 1101 | 490 | 520 | 0.325 | 0.000 |
| π_min_ | 6010101 | 6479975 | 3111026 | 3174407 | 0.096 | 0.427 |
| γ_aem_ | 3.830 | 4.000 | 1.643 | 1.733 | 0.089 | 0.529 |
| μ_em_ | 0.134 | 0.101 | 0.043 | 0.057 | 0.322 | 0.000 |
| σ | 2.033 | 2.000 | 0.543 | 0.577 | 0.084 | 0.609 |
| τ_exh_ | 5.879 | 5.900 | 1.026 | 1.000 | 0.068 | 0.843 |
| τ_ih_ | 6.097 | 6.00 | 1.001 | 1.000 | 0.080 | 0.668 |
| α_vh_ | 0.470 | 0.500 | 0.273 | 0.289 | 0.083 | 0.621 |
| α_hv_ | 0.507 | 0.500 | 0.268 | 0.289 | 0.077 | 0.704 |
| φ | 0.286 | 0.337 | 0.164 | 0.177 | 0.207 | 0.002 |
| β_2013_ | 565 | 545 | 4.430 | 14.428 | 0.684 | 0.000 |
| β_2014_ | 864 | 878 | 7.012 | 14.430 | 0.507 | 0.000 |
| μ_a_ | 0.529 | 0.497 | 0.117 | 0.288 | 0.295 | 0.000 |
| μ_i_ | 0.695 | 0.496 | 0.226 | 0.288 | 0.378 | 0.000 |

**Cycle 2** narrowing down the range in Cycle 1 according to the CDF

Passing rate: 48/200,000 = 0.474%


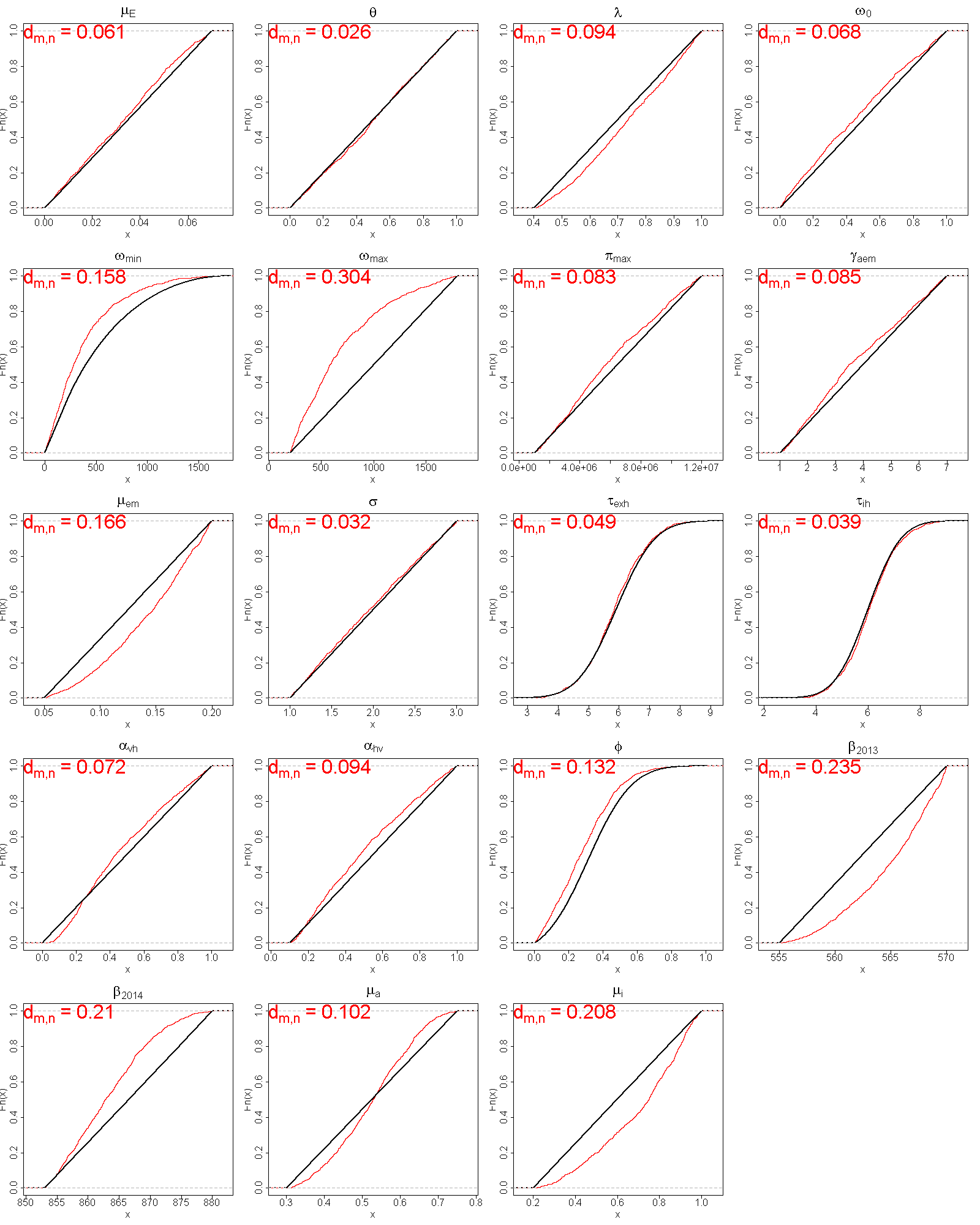


Fig S3. The CDF for pass (red) and fail (black) groups in Cycle 2

Table S7. The mean and standard deviation of pass and fail group, and the value dm,n and the p-value of Kolmogorov–Smirnov statistic in Cycle 2

|  | PassMean | FailMean | PassStd | FailStd | d_m,n_ | p-value |
| --- | --- | --- | --- | --- | --- | --- |
| μ_E_ | 0.033 | 0.035 | 0.019 | 0.020 | 0.061 | 0.002 |
| θ | 0.504 | 0.500 | 0.284 | 0.289 | 0.026 | 0.527 |
| λ | 0.733 | 0.700 | 0.163 | 0.173 | 0.094 | 0.000 |
| ω_0_ | 0.458 | 0.500 | 0.289 | 0.288 | 0.068 | 0.000 |
| ω_min_ | 376 | 501 | 326 | 393 | 0.158 | 0.000 |
| ω_max_ | 697 | 1002 | 401 | 462 | 0.304 | 0.000 |
| π_min_ | 6059966 | 6482097 | 3081708 | 3177941 | 0.083 | 0.000 |
| γ_aem_ | 3.790 | 3.995 | 1.730 | 1.730 | 0.085 | 0.000 |
| μ_em_ | 0.141 | 0.125 | 0.039 | 0.043 | 0.166 | 0.000 |
| σ | 1.969 | 2.001 | 0.580 | 0.577 | 0.032 | 0.281 |
| τ_exh_ | 5.849 | 5.904 | 0.963 | 1.001 | 0.049 | 0.022 |
| τ_ih_ | 6.060 | 5.998 | 1.009 | 1.000 | 0.039 | 0.109 |
| α_vh_ | 0.477 | 0.500 | 0.264 | 0.289 | 0.072 | 0.000 |
| α_hv_ | 0.505 | 0.550 | 0.251 | 0.260 | 0.094 | 0.000 |
| φ | 0.285 | 0.338 | 0.175 | 0.177 | 0.132 | 0.000 |
| β_2013_ | 565 | 562 | 3.785 | 4.326 | 0.235 | 0.000 |
| β_2014_ | 863 | 867 | 6.306 | 7.803 | 0.210 | 0.000 |
| μ_a_ | 0.526 | 0.525 | 0.102 | 0.130 | 0.102 | 0.000 |
| μ_i_ | 0.702 | 0.598 | 0.203 | 0.230 | 0.208 | 0.000 |

**Cycle 3** narrowing down the range in Cycle 2 according to the CDF

Passing rate: 2,743/200,000 = 1.37%


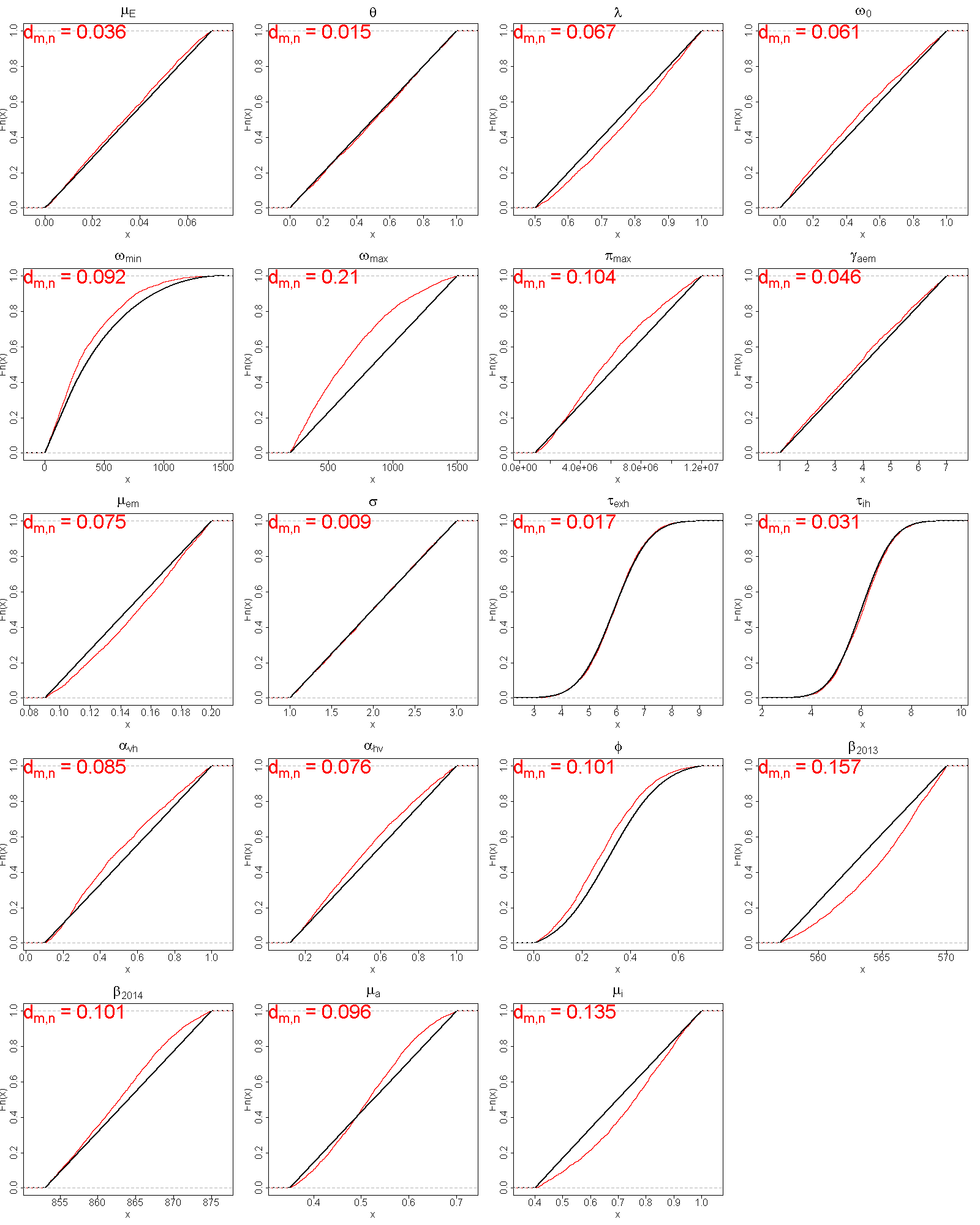


Fig S4. The CDF for pass (red) and fail (black) groups in Cycle 3

Table S8. The mean and standard deviation of pass and fail group, and the value dm,n and the p-value of Kolmogorov–Smirnov statistic in Cycle 3

|  | PassMean | FailMean | PassStd | FailStd | d_m,n_ | p-value |
| --- | --- | --- | --- | --- | --- | --- |
| μ_E_ | 0.034 | 0.035 | 0.020 | 0.020 | 0.036 | 0.002 |
| θ | 0.504 | 0.499 | 0.288 | 0.289 | 0.015 | 0.594 |
| λ | 0.771 | 0.750 | 0.140 | 0.145 | 0.067 | 0.000 |
| ω_0_ | 0.467 | 0.501 | 0.288 | 0.288 | 0.061 | 0.000 |
| ω_min_ | 358 | 424 | 286 | 327 | 0.092 | 0.000 |
| ω_max_ | 672 | 850 | 336 | 375 | 0.210 | 0.000 |
| π_min_ | 5946694 | 6502586 | 2954953 | 3177213 | 0.104 | 0.000 |
| γ_aem_ | 3.860 | 4.002 | 1.718 | 1.731 | 0.046 | 0.000 |
| μ_em_ | 0.150 | 0.145 | 0.031 | 0.032 | 0.075 | 0.000 |
| σ | 1.997 | 1.998 | 0.573 | 0.577 | 0.009 | 0.971 |
| τ_exh_ | 5.889 | 5.899 | 0.972 | 1.001 | 0.017 | 0.448 |
| τ_ih_ | 6.033 | 5.997 | 0.997 | 1.002 | 0.031 | 0.011 |
| α_vh_ | 0.511 | 0.551 | 0.251 | 0.260 | 0.085 | 0.000 |
| α_hv_ | 0.523 | 0.561 | 0.246 | 0.254 | 0.076 | 0.000 |
| φ | 0.287 | 0.321 | 0.150 | 0.153 | 0.101 | 0.000 |
| β_2013_ | 565 | 563 | 3.490 | 3.753 | 0.157 | 0.000 |
| β_2014_ | 863 | 864 | 5.804 | 6.345 | 0.101 | 0.000 |
| μ_a_ | 0.516 | 0.525 | 0.087 | 0.101 | 0.096 | 0.000 |
| μ_i_ | 0.742 | 0.699 | 0.158 | 0.173 | 0.135 | 0.000 |

**Cycle 4** narrowing down the range in Cycle 3 according to the CDF

Passing rate: 2,863/100,000 = 2.86%


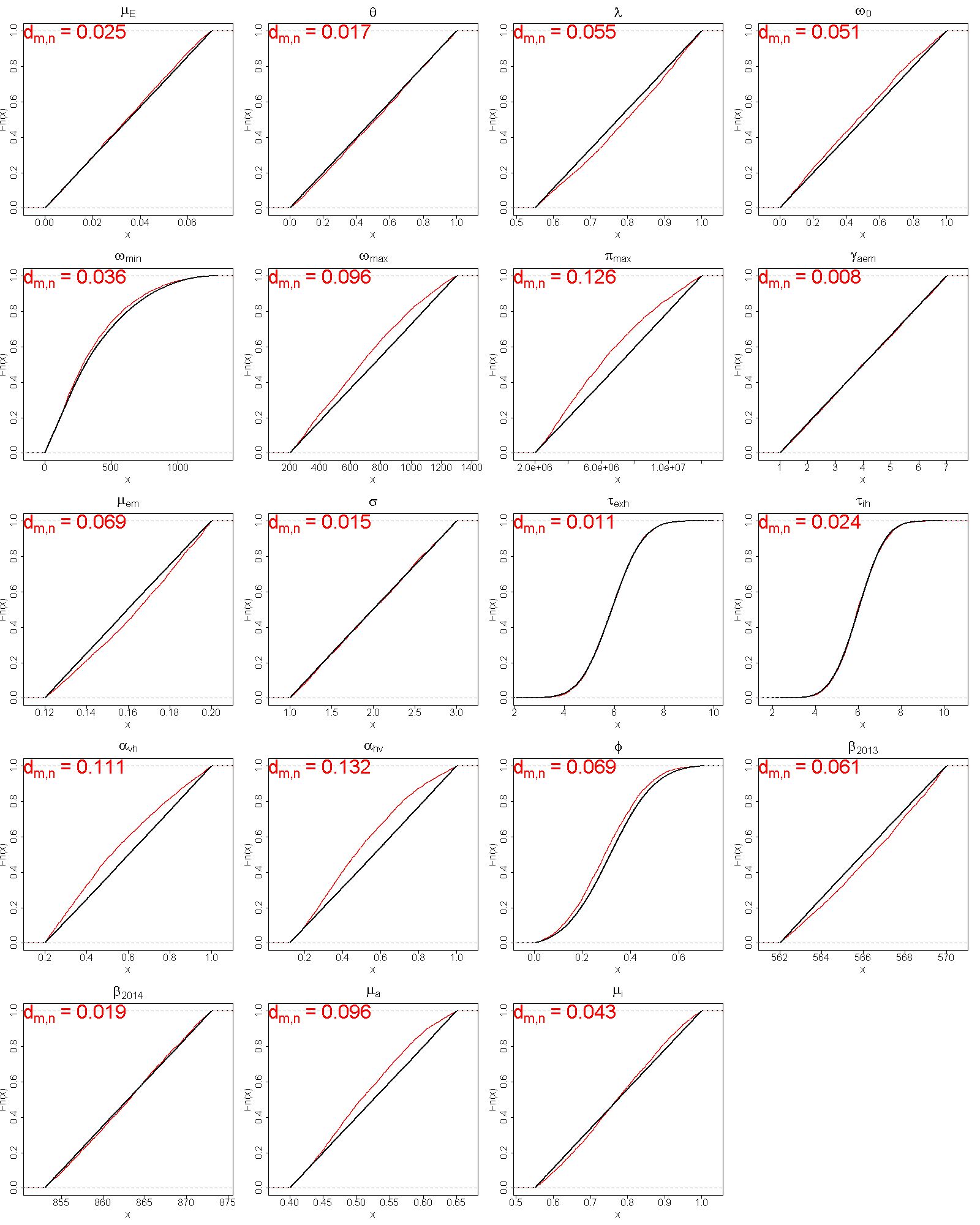


Fig S5. The CDF for pass (red) and fail (black) groups in Cycle 4

Table S9. The mean and standard deviation of pass and fail group, and the value dm,n and the p-value of Kolmogorov–Smirnov statistic in Cycle 4

|  | PassMean | FailMean | PassStd | FailStd | d_m,n_ | p-value |
| --- | --- | --- | --- | --- | --- | --- |
| μ_E_ | 0.034 | 0.035 | 0.020 | 0.020 | 0.025 | 0.0650 |
| θ | 0.509 | 0.501 | 0.284 | 0.289 | 0.017 | 0.377 |
| λ | 0.789 | 0.775 | 0.129 | 0.130 | 0.055 | 0.000 |
| ω_0_ | 0.473 | 0.501 | 0.284 | 0.289 | 0.051 | 0.000 |
| ω_min_ | 354 | 375 | 272 | 284 | 0.036 | 0.001 |
| ω_max_ | 688 | 751 | 302 | 318 | 0.096 | 0.000 |
| π_min_ | 6266253 | 7022680 | 2735351 | 2885491 | 0.126 | 0.000 |
| γ_aem_ | 4.010 | 4.003 | 1.732 | 1.734 | 0.008 | 0.990 |
| μ_em_ | 0.163 | 0.160 | 0.023 | 0.023 | 0.069 | 0.000 |
| σ | 2.000 | 1.999 | 0.573 | 0.579 | 0.015 | 0.548 |
| τ_exh_ | 5.900 | 5.902 | 0.983 | 1.001 | 0.011 | 0.917 |
| τ_ih_ | 6.000 | 6.000 | 0.997 | 0.997 | 0.024 | 0.076 |
| α_vh_ | 0.548 | 0.602 | 0.227 | 0.230 | 0.111 | 0.000 |
| α_hv_ | 0.494 | 0.562 | 0.232 | 0.254 | 0.132 | 0.000 |
| φ | 0.297 | 0.318 | 0.136 | 0.139 | 0.069 | 0.000 |
| β_2013_ | 566 | 566 | 2.296 | 2.307 | 0.061 | 0.000 |
| β_2014_ | 863 | 863 | 5.614 | 5.778 | 0.019 | 0.295 |
| μ_a_ | 0.512 | 0.525 | 0.067 | 0.072 | 0.096 | 0.000 |
| μ_i_ | 0.772 | 0.775 | 0.121 | 0.130 | 0.043 | 0.000 |

**Cycle 5** narrowing down the range in Cycle 4 according to the CDF

Passing rate: 5,320/100,000 = 5.32%


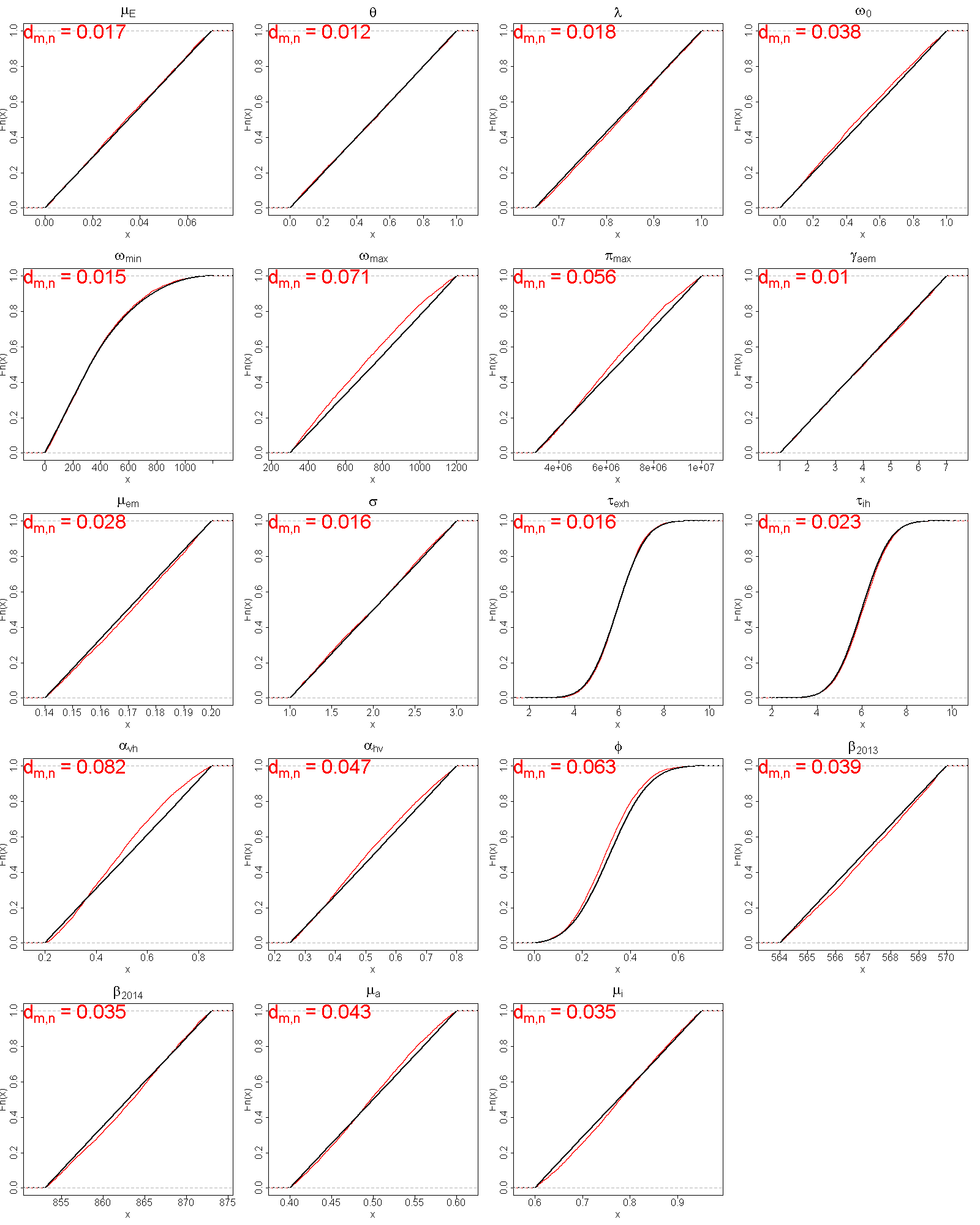


Fig S6. The CDF for pass (red) and fail (black) groups in Cycle 5

Table S10. The mean and standard deviation of pass and fail group, and the value dm,n and the p-value of Kolmogorov–Smirnov statistic in Cycle 5

|  | PassMean | FailMean | PassStd | FailStd | d_m,n_ | p-value |
| --- | --- | --- | --- | --- | --- | --- |
| μ_E_ | 0.035 | 0.035 | 0.020 | 0.020 | 0.017 | 0.119 |
| θ | 0.499 | 0.501 | 0.291 | 0.288 | 0.012 | 0.471 |
| λ | 0.828 | 0.825 | 0.100 | 0.101 | 0.018 | 0.072 |
| ω_0_ | 0.482 | 0.502 | 0.286 | 0.289 | 0.038 | 0.000 |
| ω_min_ | 370 | 375 | 256 | 264 | 0.015 | 0.214 |
| ω_max_ | 709 | 752 | 252 | 260 | 0.071 | 0.000 |
| π_min_ | 6314976 | 6508107 | 1927820 | 2029526 | 0.056 | 0.000 |
| γ_aem_ | 4.012 | 4.000 | 1.743 | 1.734 | 0.010 | 0.650 |
| μ_em_ | 0.171 | 0.170 | 0.017 | 0.017 | 0.028 | 0.001 |
| σ | 1.987 | 2.004 | 0.577 | 0.578 | 0.016 | 0.143 |
| τ_exh_ | 5.891 | 5.898 | 0.966 | 1.003 | 0.016 | 0.135 |
| τ_ih_ | 6.031 | 5.996 | 1.002 | 1.001 | 0.023 | 0.009 |
| α_vh_ | 0.503 | 0.526 | 0.170 | 0.188 | 0.082 | 0.000 |
| α_hv_ | 0.513 | 0.526 | 0.153 | 0.159 | 0.047 | 0.000 |
| φ | 0.299 | 0.316 | 0.121 | 0.126 | 0.063 | 0.000 |
| β_2013_ | 567 | 567 | 1.718 | 1.729 | 0.039 | 0.000 |
| β_2014_ | 863 | 863 | 5.589 | 5.782 | 0.035 | 0.000 |
| μ_a_ | 0.498 | 0.500 | 0.055 | 0.058 | 0.043 | 0.000 |
| μ_i_ | 0.778 | 0.775 | 0.096 | 0.101 | 0.035 | 0.000 |

The passing rate and the range for each parameter were summarized in the following table.

Table S11. Passing rate and parameter range for 5 cycles

|  | Cycle 1 | Cycle 2 | Cycle 3 | Cycle 4 | Cycle 5 |
| --- | --- | --- | --- | --- | --- |
| Passing rate | 0.0104% | 0.474% | 1.37% | 2.86% | 5.32% |
| μ_E_ | 0-0.1 | 0-0.07 | 0-0.07 | 0-0.07 | 0-0.07 |
| θ | 0-1 | 0-1 | 0-1 | 0-1 | 0-1 |
| λ | 0-1 | 0.4-1 | 0.5-1 | 0.55-1 | 0.65-1 |
| ω_0_ | 0-1 | 0-1 | 0-1 | 0-1 | 0-1 |
| ω_min_ | 0-ω_max_ | 0-ω_max_ | 0-ω_max_ | 0-ω_max_ | 0-ω_max_ |
| ω_max_ | 200-2,000 | 200-1,800 | 200-1,500 | 200-1,300 | 300-1,200 |
| π_max_ | 1.0*10^6^-1.2*10^7^ | 1.0*10^6^-1.2*10^7^ | 1.0*10^6^-1.2*10^7^ | 2.0*10^6^-1.2*10^7^ | 3*10^6^-1.0*10^7^ |
| γ_aem_ | 1-7 | 1-7 | 1-7 | 1-7 | 1-7 |
| μ_em_ | 0-0.2 | 0.05-0.2 | 0.09-0.2 | 0.12-0.2 | 0.14-0.2 |
| σ | 1-3 | 1-3 | 1-3 | 1-3 | 1-3 |
| τ_exh_ | 3-9 | 3-9 | 3-9 | 3-9 | 3-9 |
| τ_ih_ | 3-9 | 3-9 | 3-9 | 3-9 | 3-9 |
| α_vh_ | 0-1 | 0-1 | 0.1-1 | 0.2-1 | 0.20-0.85 |
| α_hv_ | 0-1 | 0.1-1 | 0.12-1 | 0.12-1 | 0.25-0.80 |
| φ | 0-1 | 0-1 | 0-0.7 | 0-0.7 | 0-0.7 |
| β_2013_ | 520-570 | 555-570 | 557-570 | 562-570 | 564-570 |
| β_2014_ | 853-903 | 853-880 | 853-875 | 853-873 | 853-873 |
| μ_a_ | 0-1 | 0.3-0.75 | 0.35-0.7 | 0.4-0.65 | 0.4-0.6 |
| μ_i_ | 0-1 | 0.2-1 | 0.4-1 | 0.55-1 | 0.6-0.95 |

# Events and transition rates of the stochastic model

ODEs were used for black events, stochastic simulations were used for blue ones. The functions for temperature- and density-dependent rates were listed in Section 2. The ID of the Event is the same as in Fig 2 of the main text.

Table S12. Events, effects and the transition rates in the stochastic model

| Event | Effect | Transition rate |
| --- | --- | --- |
| 1. Egg death | E -> E - 1 | $\omega_{1}=\mu_{E}E$ |
| 1. Egg hatching | E -> E – 1  L -> L +1 | $\omega_{2}={\kappa f}_{E}E$ |
| 1. Larval death | L -> L – 1 | $\omega_{3}=m_{L}L$ |
| 1. Pupation | L -> L – 1  P -> P + 1 | $\omega_{4}=f_{L}L$ |
| 1. Pupal death | P -> P – 1 | $\omega_{5}={(m}_{P}+(1-\frac{1}{2}e^{-\mu_{em}(1+\frac{P}{LReal})})f_{P})P$ |
| 1. Adult emergence | P -> P – 1  Aeu-> Aeu + 1 | $\omega_{6}={{\frac{1}{2}e}^{-\mu_{em}(1+\frac{P}{LReal})}f}_{P}P$ |
| 1. Emerging adult death | Aeu -> Aeu – 1 | $\omega_{7}=m_{A}Aeu$ |
| 1. Taking the first blood meal | Aeu -> Aeu – 1  As -> As + 1 | $\omega_{8}=1/\gamma_{aem}Aeu$ |
| 1. Susceptible mosquito death | As -> As - 1 | $\omega_{9}=m_{A}As$ |
| 1. Oviposition by susceptible mosquito | E -> E +1 | $\omega_{10}={n_{e}f}_{ag}As$ |
| 1. Oviposition by exposed mosquito | E -> E +1 | $\omega_{11}={n_{e}f}_{ag}Ae$ |
| 1. Infection via human contagion | As -> As – 1  Ae -> Ae + 1 | $\omega_{12}=b\alpha_{hv}\frac{Hi}{N}As$ |
| 1. Exposed mosquito death | Ae -> Ae - 1 | $\omega_{13}=m_{A}Ae$ |
| 1. Exposed mosquito becoming infectious | Ae -> Ae – 1  Ai -> Ai + 1 | $\omega_{14}=f_{exv}Ae$ |
| 1. Oviposition by infectious mosquitoes | E -> E +1 | $\omega_{15}=n_{e}f_{ag}Ai$ |
| 1. Infectious adult death | Ai -> Ai – 1 | $\omega_{16}={\sigma m}_{A}Ai$ |
| 1. Susceptible human birth | Hs -> Hs + 1 | $\omega_{17}=\alpha_{H}N$ |
| 1. Susceptible human death | Hs -> Hs – 1 | $\omega_{18}=\mu_{H}Hs$ |
| 1. Human infection via mosquito bite | Hs -> Hs -1  He -> He + 1 | $\omega_{19}=b\alpha_{vh}\frac{Ai}{N}Hs$ |
| 1. Exposed human death | He -> He – 1 | $\omega_{20}=\mu_{H}He$ |
| 1. Exposed human becoming infectious | He -> He – 1  Hi -> Hi + 1 | $\omega_{21}=1/\tau_{exh}He$ |
| 1. Infectious human death | Hi -> Hi – 1 | $\omega_{22}=\mu_{H}Hi$ |
| 1. Human recovery | Hi -> Hi – 1  Hr -> Hr + 1 | $\omega_{23}={1/\tau}_{ih}Hi$ |
| 1. Recovered human death | Hr -> Hr - 1 | $\omega_{24}=\mu_{H}Hr$ |

# 5. The hybrid deterministic/stochastic model with an adaptive tau-leap algorithm

The state variable at time t is denoted as X(t) = (E(t), L(t), P(t), Aeu(t), As(t), Ae(t), Ai(t), Hs(t), He(t), Hi(t), Hr(t)). According to the transition rates, the 24 events are partitioned into two sets, E_s_ for slow events (10 slow events as Events 12-14, 16, and 19-24), and E_f_ for fast events (14 fast events as Events 1-11, 15, 17 and 18). The transition rate for events E_si_ is denoted as ω_si_, and for events E_fj_ as ω_fj_. Let M_si_(t) and M_fj_(t) represent the number of times slow events i and fast events j happen by time t, respectively.

Initialize: set time t to 1and set the initial state variable to X_0_

(1) Calculate the value for E, L, P, Aeu, As, and Hs by using ODEs before the infected case was imported to Guangzhou at Day k. Then round the results to integer and add 1 to Hi to represent the introduction of the imported case. Set t to k. (Since there are plenty of human and mosquito in this time period from Day 1 to Day k and the dengue virus has not been introduced to the system yet, stochasticity plays little role here and deterministic model was used to save time.)

(2) Set the time step τ as 1/5.

(3) For slow events, calculate the integrated transition rate $R_{si}= \int_{t}^{t+\tau} w_{si}\left( t \right)dt$ (i = 1, 2, …, 10). Because of the small time increment, the number of times each event happens in this small time interval δM_si_ = M_si_(t + τ) - M_si_(t) is proximately Poisson distribution. Thus we calculate δM _si_ ≈ Poisson (R_si_). The change of each state variable δX_p_ (p = 6-7, 9-11) is calculated from the number of times each slow event happens in this time period. _j_ If any X_p_ +δX_p_ < 0, then τ = τ/2 and repeat step 3; else X_p_ = X_p_ +δX_p_*.*

(4) For fast state variables, use deterministic model to calculate the new value at time t + τ. Set t = t + τ.

(5) If t < 1096 (Dec 31^st^, 2014), go to step 2.

# References

1. Soetaert K, Petzoldt T, Setzer RW. Solving differential equations in R: package deSolve. J Stat Softw. 2010;33.

2. Karl S, Halder N, Kelso JK, Ritchie SA, Milne GJ. A spatial simulation model for dengue virus infection in urban areas. BMC Infect Dis. 2014;14(1):447.

3. Huang E, Wu Z. Biological characteristics and seasonal abundance of Aedes albopictus. Journal of Fujian Agriculture and Forestry University (Natural Science Edition). 2006;3: 246-250. (in Chinese)

4. Statistical Bureau of Guangdong. Guangdong statistical yearbook 2012. Beijing: China Statistical Publishing House; 2012. (in Chinese)

5. Statistical Bureau of Guangdong. Guangdong statistical yearbook 2013. Beijing: China Statistical Publishing House; 2013. (in Chinese)

6. Statistical Bureau of Guangdong. Guangdong statistical yearbook 2014. Beijing: China Statistical Publishing House; 2014. (in Chinese)

7. Liu Z, Zhang Y, Yang Y. Population dynamics of Aedes (Stegomyia) albopictus (skuse) under laboratory conditions. Acta Entomologica Sinica. 1985;28: 274-280. (in Chinese)

8. Delatte H, Gimonneau G, Triboire A, Fontenille D. Influence of temperature on immature development, survival, longevity, fecundity, and gonotrophic cycles of Aedes albopictus, vector of chikungunya and dengue in the Indian Ocean. J Med Entomol. 2009;46: 33-41.

9. Tran A, L'Ambert G, Lacour G, Benoît R, Demarchi M, Myriam C, et al. A rainfall-and temperature-driven abundance model for Aedes albopictus populations. Int J Environ Res Publ Health. 2013; 10: 1698-1719.

10. Li B. Studies on Gonotrophic Rhythm, Gonotrophic Cycle, and Parity of Aedes albopictus. Zhongguo Mei Jie Sheng Wu Xue Ji Kong Zhi Za Zhi. 1991; 7(3): 174–7. (in Chinese)

11. Li J, Zhu G, Zhou H, Tang J, Cao J. Effect of different temperatures on development of Aedes albopictus. Zhongguo Xue Xi Chong Bing Fang Zhi Za Zhi.2015 Feb; 27(1): 1–3. (in Chinese)

12. Jiang Y, Yan Z, Hu Z, Li C, Wu H. The influence of dengue virus infection upon the physiological and ecological nature of Aedes albopictus. Re Dai Yi Xue Za Zhi. 2009 Apr; 9(4): 401–2, 374. (in Chinese)

13. Chan M, Johansson MA. The incubation periods of dengue viruses. PLos One. 2012 Nov 30; 7(11): e50972 doi: 10.1371/journal.pone.0050972.

14. Liang F. Virological and Serological Surveillance of Dengue Fever in Guangdong 1992~ 1995. Ji Bing Jian Ce. 1997; 12(11): 405–8. (in Chinese)

15. Sharpe PJ, DeMichele DW. Reaction kinetics of poikilotherm development. J Theor Biol. 1977 Feb 21; 64(4): 649-70.

16. Zhong Z, He G. The life table of laboratory Aedes albopictus under various temperatures. Zhongshan Yi Ke Da Xue Xue Bao. 1988;9(3):35-9. (in Chinese)

17. Beven K, Freer J. Equifinality, data assimilation, and uncertainty estimation in mechanistic modelling of complex environmental systems using the GLUE methodology. J Hydrol. 2001;249(1–4):11-29.

18. Cheng Q, Jing Q, Spear RC, Marshall JM, Yang Z, Gong P. Climate and the timing of imported cases as determinants of the dengue outbreak in Guangzhou, 2014: Evidence from a mathematical model. PLoS Negl Trop Dis. 2016;10(2):e0004417.
